# Applying intensified design of experiments to mammalian cell culture processes

**DOI:** 10.1002/elsc.202100123

**Published:** 2021-11-24

**Authors:** Verena Nold, Lisa Junghans, Lorenzo Bisgen, Raphael Drerup, Beate Presser, Ingo Gorr, Thomas Schwab, Bettina Knapp, Stefan Wieschalka

**Affiliations:** ^1^ Development Biologicals Boehringer Ingelheim Pharma GmbH & Co KG Biberach an der Riß Germany; ^2^ University of Ulm Ulm Germany

**Keywords:** best‐cost strategy, early stage development, industrial mammalian cell culture, intensified design of experiments, modeling upstream bioprocessing

## Abstract

The analysis of data collected using design of experiments (DoE) is the current gold standard to determine the influence of input parameters and their interactions on process performance and product quality. In early development, knowledge on the bioprocess of a new product is limited. Many input parameters need to be investigated for a thorough investigation. For eukaryotic cell cultures, intensified DoE (iDoE) has been proposed as efficient tool, requiring fewer bioreactor runs by introducing setpoint changes during the bioprocess. We report the first successful application of iDoE to mammalian cell culture, performing sequential setpoint changes in the growth phase for the selected input parameters temperature and dissolved oxygen. The process performance data were analyzed using ordinary least squares regression. Our results indicate iDoE to be applicable to mammalian bioprocesses and to be a cost‐efficient option to inform modeling early on during process development. Even though only half the number of bioreactor runs were used in comparison to a classical DoE approach, the resulting models revealed comparable input‐output relations. Being able to examine several setpoint levels within one bioreactor run, we confirm iDoE to be a promising tool to speed up biopharmaceutical process development.

**Abbreviations**
CHO: Chinese hamster ovary
DO: dissolved oxygen
DoE: design of experiments
ESD: early stage development
iDoE: intensified DoE
IgG: immunoglobulin G
mDoE: mechanistic DoE
MPC: model predictive control
OLS: ordinary least square
PI: prediction interval
QbD: quality by design
RMSE: root mean squared error
TCD: total cell density
VCD: viable cell density

## 1 INTRODUCTION

Over the last decade, biopharmaceutical industry yielded an increasing number of novel products and faced a strong competition among individual companies to accelerate the development pipeline [4, 5]. Biopharmaceutical development primarily aims at providing products of reproducibly high quality and secondarily at lowering the costs of manufacturing [6]. Both goals can be achieved by following the quality by design (QbD) concept, which is aiming for a deeper understanding of the product and its underlying manufacturing process. Thus, biopharmaceutical industry spent significant efforts to move from a quality by testing approach to a systematic QbD strategy [7]. The gathered knowledge on the influence of input parameters on process performance as output parameters may subsequently be used for the optimization of the upstream bioprocess on the one hand and building of models on the other. Valid models are a prerequisite for accurate predictions and thus for the determination of input parameter optima.

**PRACTICAL APPLICATION**

Intensified design of experiments (iDoE) is an emerging approach used for efficient data acquisition to inform modelling. Several publications showed that for *Escherichia coli* bioprocesses the use of intra‐experimental setpoint changes allows to decrease the number of experiments needed to build predictive models [1, 2, 3]. We show that the application of iDoE to the growth phase of mammalian cell culture processes is technically and biologically possible. Intriguingly, iDoE requires only 50% of the bioreactors used in a classical DoE to determine and model similar input‐output relations. The iDoE approach therefore appears to be a promising best‐cost approach to increase process understanding, especially if little prior knowledge exists and resources are limited, as in early stage bioprocess development.

To build predictive process models in a cost‐efficient way, the use of experimental designs that are tailored to the research question and practical constraints is implied. Suitedness of designs may a priori be evaluated with respect to their capability to detect effects relative to the uncertainty of the method, to disentangle effects from potentially confounding effects, and regarding their robustness against missing data. Design of experiments (DoE) approaches based on optimality criteria are one option to obtain designs that allow for studying the influence of input parameters (factors or conditions that are directly adjustable to a setpoint and under control throughout the process) on output parameters (read outs which cannot be directly adjusted but are a consequence of the input parameters applied, and which serve as indicator for process performance or product quality [PQ]) in a multivariate manner, while still being affordable and informative for models that are predicting outcomes of future batches [8]. Using fixed process parameter setpoints for each individual bioreactor run, DoE are well suited to elucidate static relationships between input parameters, process performance and PQ [3, 7]. Classical use cases of DoE in the bioproduction environment are for example scale‐down models [9], validation of control ranges of control parameters [10], and static optimization [11, 12, 13]. Typically, DoE implements response surface modeling [11, 14].

Since biological systems are sensitive to a plethora of input parameters, the investigation of a large variety of parameters, at different levels, and in multiple combinations would be needed to address the high cellular complexity. Even though classical DoE is capable of allocating several input parameters, the number of bioreactor runs needed to ensure sufficient power to detect all possible main and higher‐order effects without the use of prior knowledge would be technically impractical. Screening approaches to reduce the number of eligible input parameters are one option to overcome this limitation, however at the risk of missing relevant interactions. The sequential addition of further experiments through augmentation approaches could address potentially missing interaction effects, but is time‐consuming and less efficient than planning for them upfront, since additional degrees of freedom need to be spent to account for potential block effects attributable to the different seed trains needed. Model‐based DoE (mDoE) is a special approach to select those combinations of setpoint levels which are most probable to extend the existing model, since mDoE incorporates mechanistic knowledge during the planning phase [15, 16, 17]. To integrate newly generated data, often mechanistic modeling is used [18, 19]. However, detailed prior knowledge in the shape of (mechanistic) process models is usually not available during early stage development (ESD), which limits the applicability of mDoE.

In 2016, von Stosch et al. investigated the idea of applying targeted intra‐experimental setpoint changes of process parameter setpoints over the course of single bioreactor runs [2]. The concept, known as “intensified Design of Experiments” (iDoE), was successfully applied to *Escherichia coli* processes, extending the “classical DoE” framework (abbreviated by DoE in the following) [1, 3, 7]. Allowing several combinations of setpoints levels within a single bioreactor run enables iDoE to cover the same design space as DoE, but with less experimental runs [1]. However, intra‐experimental setpoint changes could seriously impact complex mammalian cells, by triggering metabolic shifts or irreversible alterations that may even lead to a collapse of the whole process [20]. An application of iDoE to mammalian cell culture processes was thus discussed to be potentially detrimental to the bioprocess [7].

The goal of this work was to determine the applicability of iDoE to an industrial biopharmaceutical production process for an immunoglobulin G4 (IgG4) mAb, using Chinese hamster ovary (CHO) cells. We hypothesized, that the application of iDoE is biologically feasible for mammalian cell culture processes with certain adjustments (i) and that similar insights into the input‐output relations as with data from classical DoE are obtainable using iDoE (ii), while needing less bioreactor runs (iii). Leveraging the scheduled changes in setpoint levels within bioreactor runs of the iDoE, a more far reaching goal was to provide first insights into the role of time on the effects of the input parameters.

## 2 MATERIALS AND METHODS

### 2.1 Planning of the D‐optimal DoE

A D‐optimal design with two factors, i.e. input parameters (temperature à 5 levels and dissolved oxygen (DO) à 3 levels) accommodating 12 combinations thereof was created and evaluated in Design Expert Version 12.0.0.6. StatEase. The underlying model structure of the design contains all model terms representing main, quadratic and two factorial interaction effects. As responses (i.e. output parameters), total cell density (TCD), viable cell density (VCD), viability and glucose, as well as lactate concentration were measured. The DoE was evaluated regarding the power to detect effects of these model terms when assuming effect sizes of three relative to the noise of measurement imprecision and setting the significance level to 5% for the named effects. To mimic the situation in ESD, no prior information regarding effect sizes was included into design planning and evaluation. The correlations of model terms for the given design was evaluated in addition to power estimates and evaluated regarding their criticality. Moreover, the leverage of the planned design points was checked and the saturation of the design relative to the degrees of freedom required for the full model structure was considered during DoE evaluation.

### 2.2 Computation of the iDoE run table

Based on the above defined DoE, an iDoE has been computed as described earlier [3]. In short, the run table of the planned DoE was used as a basis for a binary optimization problem with eight constraints to compute the combinations of input parameter setpoint levels in the iDoE. The iDoE was restricted to the growth phase of the bioprocess, i.e. until day 6. After day 6, until harvest at day 14, all setpoint levels were fixed at platform center points. The iDoE has been computed using Matlab R2019a (9.6.0) using 3 stages within 6 iDoE runs. The settings of the iDoE algorithm [3] are given by $s_{k}=1$ if k is a replicate run of the classical DoE (k = {3,4,8,9,10,11}) and s =3 otherwise. The parameter *t* was set to [2 2 2]. The maximum change from one stage to the next was limited to 1 for temperature and 0.5 at minimum for both parameters DO and temperature using coded setpoint levels for DO and temperature ranging from −1 to 1.

### 2.3 Cell culture

A monoclonal CHO‐K1 GS cell line producing an IgG4 mAb was cultivated in suspension using chemically defined media and feeds (proprietary, Boehringer Ingelheim Pharma GmbH & Co. KG, Ingelheim, Germany). DoE and iDoE experiments were conducted in fed‐batch mode in 3 L glass bioreactors (proprietary bioreactor, Boehringer Ingelheim Pharma GmbH & Co. KG, Ingelheim, Germany). DO and pH were controlled using online pH (VisiFerm DO 325, Hamilton, Bonaduz, Switzerland) and DO sensors (EasyFerm Plus PHI MS 325, Hamilton, Bonaduz, Switzerland) throughout the 14 days process. Temperature and DO setpoint levels were fixed to or changed according to the planned DoE and iDoE, respectively. Feed medium was added with a constant rate, while glucose was added as bolus on a day‐to‐day basis to maintain an optimal concentration.

### 2.4 Process analytics

Online process data, i.e. pH and CO_2_, were automatically logged using a custom process management system. Routine culture samples were drawn daily over the entire process duration. Additional sampling was performed for the iDoE setups from day 0 to 4. Viability, TCD, and VCD were measured using an automated cell counter (Cedex, Roche, Basel, Switzerland), based on a white/dark image classification following trypan blue exclusion staining. Metabolites (e.g. glucose and lactate) were measured in cell free samples, using photometric assays combined in an automated wet chemical analyzer (Konelelab Prime 60i, Thermo Fisher Scientific, Waltham, MA, USA).

### 2.5 Statistical data analysis

Processing, visualization, and analysis of data was performed using R version 4.0.2. The data for DoE and iDoE were coded between −1 and +1 for input parameters and normalized between 0 and 1 for the measured output parameters of process performance. For the iDoE, the measurements within each stage were rebased by subtracting the start value of the respective stage to account for potential offsets originating from previous input setpoint. Ordinary least square models (OLS) of up to three‐factorial interactions were fitted for each output parameter using a bidirectional selection of model terms optimizing the corrected Akaike information criteria [21]. Fitting second order models, as originally planned, was underfitting the data and interaction effects of times were identified being crucial. Model validity was assessed using the amount of explained variance (*R^2^*) and *R^2^_adjusted_* in comparison to the *R^2^_predicted_* as well as the root mean squared error (*RMSE*) [22].

## 3 RESULTS

### 3.1 Design space coverage and quality of iDoE and DoE

**FIGURE 1:**
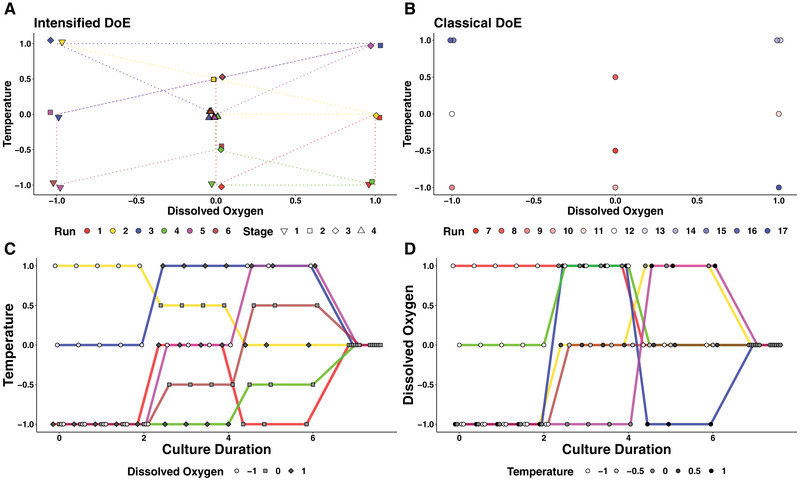
Visualization of the conducted iDoE versus DoE input factor combinations. Coverage of the design space defined by temperature and DO with the experimental runs of the iDoE (A) and DoE (B). Changes of temperature (C) and DO (D) over the first week of cell culture in the iDoE runs. Visualization of the changes (on days 2, 4 and 6) were jittered to reduce overlap for display

The iDoE resulting from the published algorithm [3] to investigate the effects of DO and temperature as input parameters during the growth phase of the cell culture process was compared with the D‐optimal DoE obtained with a commercial software. Three setpoint levels, spanning a broad range, were investigated for DO, while the effects of temperature are assessed with five different setpoint levels over a narrower range. Coverage of the design space using the iDoE approach was comparable to the coverage using DoE (Figure 1). In the iDoE bioreactor runs, temperature and DO setpoints for all runs were moved to the centre level of the design space at culture day 6. Thus, the predictive capacity of the models for output parameters indicated by process performance parameters based on iDoE data is limited to the growth phase. For the DoE, all input parameters remained constant at the initial setpoint level over the entire duration of cultivation. For the iDoE, the scheduled changes in DO and temperature setpoints resulted in the division into stages. The distribution of setpoint levels across the different stages of the iDoE was not perfectly balanced since more bioreactor runs started at the low setpoint level of temperature (Figure 1C) and DO (Figure 1D).

Since in the iDoE the setpoints for the input parameters are changed to different levels at different time points, the input parameter time was *post hoc* included into the full model of DoE and iDoE. By including the interaction of up to third order effects of time with DO as well as with temperature, the full model structure was extended to up to three‐factorial interactions. An overview of the design evaluation is provided in the supplements (Supplement 1). The power in the DoE for this modified full model structure was above 99% making the few absolute correlations above 0.4 among model terms negligible. The leverage of the individual data points decreased to 0.2 on average. Since time and stages would be fully correlated, the evaluation of design quality was carried out individually for each stage of the iDoE. While the power at a signal‐to‐noise ratio of three to detect quadratic effects of the input parameters DO and temperature was above 99%, the power to detect effects associated with the other model terms was low for stage 1 and 2. For stage 3, the power to detect quadratic effects was around 30% assuming a relative effect size of 3, and above 90% assuming a relative effect size of 10. The low power to discriminate effects in the stages of the iDoE was reflected in correlations among the model terms. The average leverage of data points for stage 1, 2, and 3 were 0.4, 0.5 and 0.7, respectively.

### 3.2 Similar coverage of process performance variability in iDoE and DoE

**FIGURE 2:**
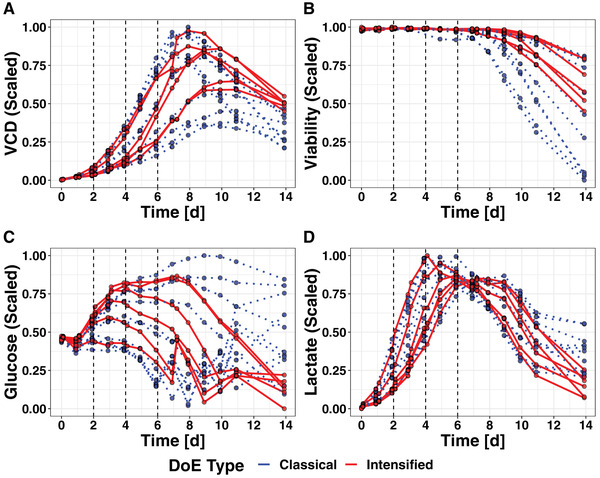
Measured process performance related outputs over the full time course. Dashed black lines indicate the timing of setpoint changes for the iDoE runs. The measurements of viable cell density (A), viability (B), glucose (C) and lactate (D) for DoE (blue) and iDoE (red) are connected with straight lines to indicate the belonging to the different bioreactors

To monitor performance of the cell culture processes, the chosen output parameters were determined on a day‐to‐day basis for DoE and iDoE, with an additional second sampling being performed for the iDoE on day 1, 2, 3 and 4. An overview on the measured process indicators VCD, viability, lactate and glucose used as output parameters is given in Figure 2. For one bioreactor run within the DoE, the stirrer stopped and caused a drop in DO and viability. Therefore, all data of this run was excluded from the analyses. *Post hoc* analyses of the design quality did not indicate critical impairment of the design, which thus was deemed to still be suitable for analysis even with one missing run (data not shown).

Data from both designs covered a broad range of output parameter values. The variability in the data allows for identifying associations between process parameters as input parameters and process performance as the output parameter. Based on looking at the trajectories, inclusion of time as necessary input parameter to describe the changes between the measurements was confirmed. Neither aberrant cell culture performance nor a complete collapse of individual iDoE bioreactor runs was detected. Critical memory effects impacting cell culture performance could also not be identified, since the cells always reacted to an expected extend and in a similar time frame despite of serval setpoint changes. All iDoE setpoints were changed to the same center level on culture day 6, thus differences in performance after day 6 for bioreactor runs of the iDoE are attributable to the sum of differences introduced during the first cultivation days. Nevertheless, with the same setpoint level an approximation in process performance was seen from day 6 to the end of process.

### 3.3 Models fit to measurements from iDoE

**FIGURE 3:**
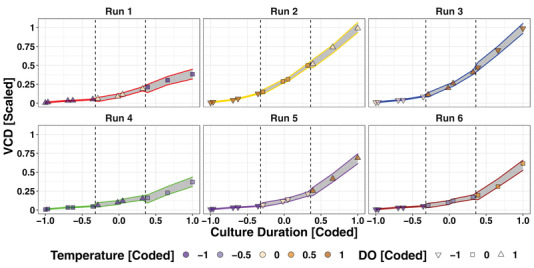
Fit of iDoE‐based regressions to VCD measurements in the iDoE. Data and the 95% confidence bands of the regressions for the individual iDoE runs over time are provided in the different windows of the figure. Levels of DO are displayed as different shapes, while temperature levels are differentiated by coloring of the shapes

Visual insights into the timely evolvement of the output parameters (process performance parameters) in dependence of temperature and DO setpoint changes in the iDoE data are provided exemplarily for VCD in Figure 3. The changing of process parameter setpoints introduced stages in the data, which are indicated by dashed lines. Bioreactor‐wise normalization to the start values of each stage was carried out to correct for potential offsets due to effects of different setpoint levels in previous stages. For each of these stages, OLS models accounting for temperature and DO as input parameters with 5 respectively 3 equidistant setpoint levels were built. Time and interactions therewith were also included in the pool of eligible model terms. Following model selection based on the Akaike information criterion corrected for the number of incorporated model terms, the quality of the models was determined. A high percentage of explained variance *R^2^adj* and a low residual root mean squared error *RMSE* relative to the methodological accuracy of the measurement was observed for the models of TCD, VCD and glucose over all stages (Table 1). The model for lactate had a low predictive propensity *R^2^_pred_* value for the latest stage of the growth phase but was well modeled during stage 1 and 2. Only few variability in viability was explained in any of the models, which is attributable to the absence of variability of viability during growth phase. The diagnostic plots including a visualization of residual error over predicted values as indicator for heteroscedasticity, quantile‐quantile plots to compare the distribution of the residuals to a Gaussian distribution, and the visualization of measured vs. predicted values (Supplement 2) for those models with good numerical quality indicators were inconspicuous. No clear pattern indicating remaining structure in the data that was not covered by the model and could explain systematic over‐ or underestimation of effects was visible. Stage‐wise predictions for each combination of temperature, DO, and time point present in the iDoE were made and the results were concatenated by adding them to the predictions from previous stages. The 95% prediction intervals become wider towards the end of the growth phase (Figure 3), which is commonly observed in culture processes due to the higher variability in the exponential phase of culture. All measured values fall within the prediction interval, indicating a valid fit of the model to the data.

**TABLE 1:** Qualities of stage‐wise models based on iDoE data

Stage	Output	*R* ^2^	*R* ^2^_adj_	*R* ^2^_pred_	RMSE	PRESS	df_num_	df_den_
1	TCD	0.99	0.99	0.98	0.05	0.15	6	23
1	VCD	0.99	0.99	0.98	0.04	0.12	8	21
1	Viability	0.16	0.13	0.06	0.43	6.11	1	28
1	Glucose	0.93	0.91	0.87	0.11	0.63	6	23
1	Lactate	1	1	0.99	0.01	0.02	9	20
2	TCD	0.99	0.99	0.98	0.14	1.46	11	12
2	VCD	0.99	0.99	0.98	0.13	1.35	11	12
2	Viability	0.44	0.39	0.27	0.28	2.54	2	21
2	Glucose	0.92	0.89	0.84	0.17	1.35	7	16
2	Lactate	0.96	0.95	0.94	0.11	0.43	5	18
3	TCD	0.99	0.99	0.95	0.32	9.74	6	11
3	VCD	0.99	0.99	0.95	0.32	9.81	6	11
3	Viability	0.53	0.43	0.1	0.18	1.08	3	14
3	Glucose	0.95	0.93	0.89	0.10	0.42	5	12
3	Lactate	0.73	0.68	0.53	0.22	1.56	3	1

Abbreviations: adj = adjusted to number of model terms; den =denominator, difference between used and eligible model terms; df = degrees of freedom; num = numerator, number of model terms used in the model; pred = prediction; PRESS = indicator of robustness of prediction in leave one out validations; R2 = coefficient of determination (explained variance); RMSE = root mean squared error of fitted to measured values.

### 3.4 Use of models obtained with iDoE data to learn about the process

**FIGURE 4:**
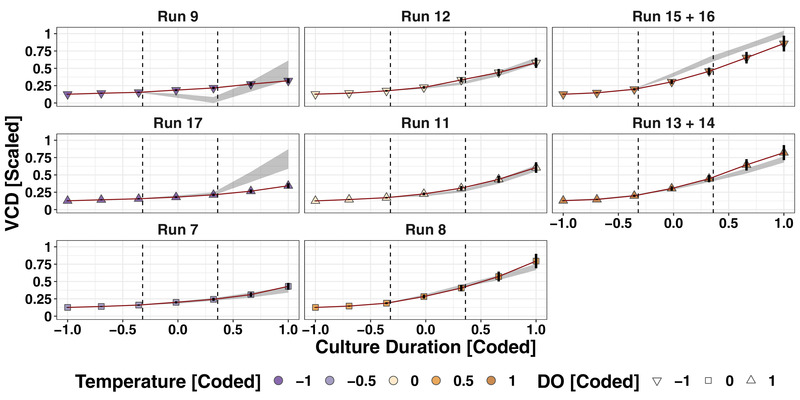
Validation of iDoE‐based models by visualization of the overlap between iDoE‐based prediction corridor with VCD measurements from DoE data. The error bars around the measurements give an impression of the historically known measurement uncertainty for VCD at the corresponding cell concentrations

The DoE data was used to externally validate the 95% prediction intervals (PIs) of the iDoE‐based models. For each combination of DO, temperature, and time of culture duration, the 95% PI was computed and compared with the measurements taken during the DoE. The overlap between iDoE‐based prediction intervals and VCD measurements from the DoE data are shown in Figure 4. The PIs for the three medium temperature levels paired with different DO setpoint levels include the measured values throughout the growth phase. However, the predictions for the lowest temperature level paired with the highest DO level overestimate the measured VCD towards the end of the growth phase (run 17). The predictions for the highest temperature level in combination with the lowest DO level suffer from overestimation (run 15 + 16), while the combination of highest temperature and highest DO indicates a trend to underestimation (run 13 + 14).

Having valid models at hand, the model coefficients (Supplement 3) can be used to compute optimal input parameter setpoints for a desired outcome. This can be done for one individual output or in a multivariate manner for several outputs and with different importance rating of the outputs or constraints to the accepted input parameter setpoints. The sign of each model term coefficient indicates whether changes in the levels of the respective model term have positive or negative effects on the output that is modelled. Importantly, all model coefficients need to be considered jointly. For example for VCD in stage 2, the interaction of the cubic effects of time with temperature has the highest positive coefficient (76.76 (β‐coefficient) ± 6.44 standard error (SE)) and the interaction of time^3^ × DO × temperature has the strongest negative coefficient (‐43.41 ± 7.21). The first indicates that VCD increased over time and that the slope is steeper the higher the temperature is. The second however adds, that the slope only increases with high temperatures in presence of medium DO levels, but not in the presence of the highest DO levels tested. This can also be seen in the middle column of Figure 4 where the last predictions for stage 2 are higher in run 8 (medium temperature, medium DO) than in run 11 and 12 (medium temperature, high or low DO, respectively).

## 4 DISCUSSION

In this first experimental case study on the applicability of iDoE to mammalian cell cultures, changes to the setpoint levels of DO and temperature were introduced at two different time points during the growth phase of the bioprocess. Concomitant effects on process performance could be modeled successfully using an adapted OLS approach, in which stage‐wise models, considering all up to three‐factorial interactions of DO, temperature and time, were concatenated. Validation of the iDoE‐based models, using data from a classical DoE performed in parallel, showed a reasonably good predictive capacity. The learnings from iDoE‐data are thus similar to learnings that can be obtained with classical DoE. It is remarkable that only with half the runs planned for classical DoE, this level of prediction was obtained. Considering direct manufacturing costs for one 500 L batch of about 0.43 million US dollars and using this as a benchmark for smaller development scales, the ecological and economical advantages of needing less bioreactors runs becomes apparent [23]. This includes a reduction of costs by decreased media demands, seed culture volumes, and the lab footprint. Data obtained using iDoE is sufficiently good to provide ESD with valuable insights into the bioprocess and implies iDoE to be a best‐cost approach that is even feasible with limited resources.

### 4.1 Considerations and requirements of iDoE for mammalian cell culture processes

To improve future iDoE user experience, lessons learned from this first application of iDoE to mammalian cell culture processes, as well as considerations regarding the limitations of iDoE, are provided in the following paragraph.

The most critical consideration is on biological feasibility. Presuming sensitivity of CHO cells to unfavorable physical parameters [20], or at least quick sequential changes, it was decided to focus on setpoint changes for temperature and DO. Since those parameters are easy to monitor and control, side effects due to equipment variability, imbalanced technical regulation parameters, or extensive reset times were mitigated. Still, it was unclear if the cells would react with aberrant and irreversible behavior in growth, metabolism, or productivity to the introduced setpoint changes. The used CHO producer cell line survived exposure to changes in both, DO and temperature at the described frequency, without a collapse of the bioprocess. The cell culture process in general not only accepted smaller and temporally limited excursions, as applied here, but also responded in an evaluable manner, that allowed us to build valid models.

The second consideration for the application of iDoE is the set of input parameters to be investigated. A prerequisite of iDoE is that the setpoints are interchangeable, as is the case for DO and temperature. However, “one‐sided factors,” such as seeding cell density or feed rate are difficult to fulfill in a normal fed batch approach and may require more complex setups, such as perfusion processes. A further prerequisite of iDoE regarding input parameters is that their effects are reversible in a sense that the system remains capable to respond to subsequent changes. This was the case in the experiments at hand. The resulting models were valid to describe the data, as judged by inspecting the prediction‐measurement accuracy, performing the standard diagnostics and by considering numerical estimates of model quality.

The third consideration for the planning of iDoE is, that reaction times of the cells or the effect size of the output parameter to the change in the input parameter may be different for each output parameter. Moreover, the effects may depend on the timing of the setpoint change in the culture process, e.g. if only few living cells are present, less intensity in the measured output parameter might be expected. Therefore, optimal sampling timepoints relative to the performed changes in setpoint levels, but also optimal timing of the changes relative to the bioprocess are to be further evaluated. Provided that mammalian cells have a slower metabolism than procaryotic cells, for each stage a duration of 2 days was planned. Within this time frame, the cells responded to setpoint changes, indicating effects of the altered input parameters. The direction of changes in output parameters due to changes in input parameters identified via the model coefficients and predictions was comparable between DoE and iDoE‐based data. However, the absolute values cannot be the same given the differences in exposure duration. Taken together, our results indicate that a shorter exposure to each setpoint level under investigation should be avoided. Before changing to the next setpoint level, sufficient time need to be provided for the biological system to (i) sense the environmental changes and (ii) to adapt in a manner that is detectable in the output parameters under investigation. On one hand, longer exposure might yield bigger effect sizes and result in a higher power to detect effects and to increase complexity of the model. On the other hand, longer exposure will be at the cost of being able to perform fewer setpoint changes, resulting in a less finer grading into stages and requiring more runs to cover the same amount of input parameter combinations than an iDoE with shorter exposure times. In sum, there is a tradeoff between making use of many setpoint changes to save experiments and to have a long enough duration of the stages to observe and determine potential effects. For application in ESD, the chosen minimal duration of 2 days appears a suited compromise. A reevaluation of these aspects during the planning of future iDoE in accordance with the scope of the respective experiments is recommended. Additionally, the latency of the equipment to adapt to the scheduled new level needs to be taken into account during the planning of the sampling frequency and the timing of setpoint changes. New physical setpoint levels are not immediately reached after adjustment of the setpoints. For example, the regulating system needs time for heating/cooling to change the temperature setpoint. This technical capability can be measured without the need of limited biomaterial. With our experimental set‐up, cells were exposed to a temperature ramp of one to two hours before the new level was reached.

Last, as for every experiment, the scope needs to be defined prior to conduction. In this study, we focused on process performance parameters as output parameters. The reversion to center setpoint level on day 6 in the iDoE bioreactor runs elucidates that effects of the growth phase are reversible in mammalian cell cultures. Potential carryover effects of differences in growth phase performance to the production phase might become apparent until process performance parameters converge under center setpoint condition. If the effects of the different growth phase setpoints would last longer into the production phase, the associated different trajectories could influence PQ‐related output parameters such as glycosylation, or fragmentation once product is formed. We did not observe such effects in a qualitative assessment of data (data not shown). To investigate effects of input parameters on PQ, the iDoE has to be carried out during the production phase.

### 4.2 Expectation of model complexity needs to be aligned with planning of the iDoE

Assuming few prior knowledge on the bioprocess and the limitation to six bioreactors, this study focused on how to best leverage limited biomaterial to efficiently obtain insights into the process characteristics of the cell line under investigation. This is a typical task in ESD. By departing from classical DoE and using OLS analyses, we not only provide a direct connection to these current gold standards in industry, but also pave the way for augmentation of the iDoE‐based findings with future experiments that usually are performed during further bioprocess development after ESD. Given that the concept of iDoE builds upon changes in setpoint levels over time, our analysis approach extends the industry standard analysis of endpoint measures by modeling the input parameters jointly with the time course. The impact of input parameter setpoints in interaction with time and even higher order terms of time in case of non‐linear evolution need to be considered for the analysis of time course data. To resolve effects of higher order model terms with a decent power under the assumption of a pre‐defined signal‐to‐noise ratio, these model terms should be included during planning of the experiment [24]. By increasing sampling frequency and dependent on the effect size, DoE designed for end point measurements may still allow for detection of interaction terms including time, as shown with the DoE included in this study. For the derived iDoE using the published algorithm [3], the statistical power to detect those was low.

Constant setpoints of input parameters as in classical DoE hinder the elucidation of effects that depend on phases of the culture, i.e. that becomes apparent as interactions with stage. Opposing effects at different time points might erase each other in an OLS model without time as (interacting) factor. Using intra‐experimental setpoint changes, iDoE may enable to investigate such timing‐dependent effects [1]. Like in DoE [24], the iDoE in this study illustrated that balancing of setpoint levels over stages is essential for the model capacities. Since the algorithm by von Stosch [3] distributes the input setpoint level combinations provided in a DoE over the stages of the iDoE without considering the correlation structure of the model terms, balance is not ensured and in our case no low temperature setpoint level in combination with high DO levels were present in later stages of this iDoE. Such imbalances or correlation among setpoint levels is likely to cause bias in the predictions of the model for the affected input combinations [24]. To mitigate effects of imbalances in the iDoE design on the models, the data was subdivided into the different stages defined by the setpoint changes, models for each stage were built individually, and the predictions were concatenated afterwards to yield predictions over the whole growth phase. The resulting models presented here were fitting well the changes in the iDoE data and were valid in predicting data obtained with classical DoE with certain limitations. The bias from the design was still visible in the prediction accuracy towards the end of the growth phase and at extreme setpoint levels.

The approach of concatenating stage‐wise models had the additional advantage to correct for offsets originating from previous setpoint levels by normalizing the changes within a stage to the respective start value. Differences in cell internal metabolic settings cannot be controlled for by this normalization within each stage. Additional output parameters dedicated to determining differences in metabolic pathways (e.g. measurement of amino acids used in the tricarboxylic acid cycle during glycolysis) would be needed to shed more light on such underlying molecular relationships.

### 4.3 Outlook: extending the usability of iDoE

In this first study on the applicability of iDoE to elucidate mammalian bioprocesses, we focused on how the process performance in the growth phase is influenced by specific input parameters. Replication of our findings regarding the feasibility of iDoE for mammalian cells in potentially more sensitive cell lines and with different input parameters and ranges is needed for confirmation. Since the ultimate goal of biopharmaceutical development is to provide patients with efficacious, affordable and save drugs, the applicability of iDoE to the production phase and throughout the bioprocess still needs to be determined. One important question future studies should address is whether intra‐experimental setpoint changes show an impact on product yield and quality. Aspects regarding the frequency of setpoint changes and sampling should be accounted for to obtain meaningful data and valid models, as discussed above. The use of iDoE could then help to define protocols for the bioprocess that contain scheduled changes of the input parameter setpoints to maximize cell growth and viability during growth phase and to optimize product formation, stability, charge patterns, and glycosylation during the production phase. Taking the concept of timed input parameter changes further, iDoE could take an additional role in the establishment of dynamic model predictive control strategies through their potential to elucidate response kinetics of the biosystem and the influence of timing and exposure to input parameter setpoint combinations on the output parameters [26]. Since the explicit description via model terms for all active effects and their higher order interactions could turn out to be highly complex, alternative analyses approaches may be considered. At the cost of exactly knowing the parameters of the model and being able to assign coefficients and the associated uncertainty ranges to them, predictions based on hybrid models or machine learning models [25] could be useful to describe the learnings from data generated with iDoE. Identification of synergies between experimental design and data analysis strategies remains an interesting task during the implementation of iDoE for industrial use.

## 5 CONCLUDING REMARKS

First application to mammalian cell culture proved that iDoE protocols allow for including transient changes in setpoints without a collapse of the upstream process. By selecting easily controllable physical input parameters whose levels were changed every other day during the first week of cell culture, we generated evidence that iDoE is technically and biologically feasible for mammalian cell culture processes. We obtained valid statistical models with iDoE data, despite conducting only about half the number of bioreactor runs than used for the simultaneously performed DoE approach. By using the same input parameters in the DoE and iDoE, we were able to validate the predictions made with the models based on iDoE data with the data from classical DoE. This is a first validation of the potential of iDoE in ESD to efficiently gain insights into the performance of mammalian cell culture processes with a fewer number of runs than the current gold standard in industry. Using the gathered knowledge to choose which experiments are needed to augment the data will furthermore aid in saving costs during late stage development. Taken together, applying iDoE in ESD of mammalian cell cultures could lay the foundation for advanced modeling and enable a more model‐driven late‐stage development.

## CONFLICT OF INTEREST

Raphael Drerup, Ingo Gorr, Lisa Junghans, Bettina Knapp, Verena Nold, Beate Presser, Thomas Schwab and Stefan Wieschalka have been employees of Boehringer Ingelheim during this study. Lorenzo Bisgen declares no conflict of interest.

## Supporting information

Supporting information.

Supporting information.

Supporting information.

## Data Availability

The data (scaled and coded) that support the findings of this study are available on request from the corresponding authors. The data are not publicly available due to privacy restrictions.
