# Supplementary material for: Applying intensified design of experiments to mammalian cell culture processes
Source: Eng Life Sci. 2021 Nov 24;22(12):784–95. doi: 10.1002/elsc.202100123 (PMC9731596; doi:10.1002/elsc.202100123)
Supplement: Supplementary file 1 — Supporting information. [file ELSC-22-784-s001.pdf]

Degrees of Freedom Full Models

iDoE Stage 1	Model	10
	Residuals	19
	Lack of Fit	14
	Pure Error	5
	Corr Total	29
iDoE Stage 2	Model	12
	Residuals	11
	Lack of Fit	11
	Pure Error	0
	Corr Total	23
iDoE Stage 3	Residuals	5
	Lack of Fit	5
	Pure Error	0
	Corr Total	17
DoE Growth Phase	Model	12
	Residuals	63
	Lack of Fit	49
	Pure Error	14
	Corr Total	75

Leverage of Design Points for Stage-Wise iDoE and DoE Full Models

iDoE Stage 1		iDoE Stage 2		iDoE Stage 3		DoE Growth Phase	
Run	Leverage	Run	Leverage	Run	Leverage	Run	Leverage
1	0.7600	1	0.4600	1	0.7452	1	0.1385
2	0.7600	2	0.6400	2	0.8331	2	0.0736
3	0.3829	3	0.7850	3	0.9090	3	0.0759
4	0.3829	4	0.7850	4	0.5558	4	0.0842
5	0.3857	5	0.7400	5	0.8585	5	0.0776
6	0.3857	6	0.6400	6	0.4793	6	0.0759
7	0.3400	7	0.3400	7	0.7296	7	0.1393
8	0.3400	8	0.3600	8	0.6245	8	0.1379
9	0.2457	9	0.4650	9	0.8837	9	0.0841
10	0.2457	10	0.4650	10	0.5436	10	0.0844
11	0.1714	11	0.4600	11	0.7699	11	0.0899
12	0.1714	12	0.3600	12	0.4724	12	0.0845
13	0.4333	13	0.3400	13	0.7545	13	0.0844
14	0.4333	14	0.3600	14	0.9583	14	0.1382
15	0.2762	15	0.4650	15	0.9242	15	0.4412
16	0.2762	16	0.4650	16	0.5632	16	0.2090
17	0.2190	17	0.4600	17	0.9116	17	0.1808
18	0.2190	18	0.3600	18	0.4835	18	0.1908
19	0.3400	19	0.4600			19	0.1830
20	0.3400	20	0.6400			20	0.2112
21	0.2457	21	0.7850			21	0.4390
22	0.2457	22	0.7850			22	0.2319
23	0.1714	23	0.7400			23	0.1159
24	0.1714	24	0.6400			24	0.1221
25	0.7600					25	0.1383
26	0.7600					26	0.1221
27	0.3829					27	0.2357
28	0.3829					28	0.2229
29	0.3857					29	0.1153
30	0.3857					30	0.1133
						31	0.1230
						32	0.1131
						33	0.1145
						34	0.2207
						35	0.2229
						36	0.1153
						37	0.1133
						38	0.1230
						39	0.1131
						40	0.1145
						41	0.2207
						42	0.3029
						43	0.1285
						44	0.1176
						45	0.1296

						46	0.1176
						47	0.1286
						48	0.3027
						49	0.3029
						50	0.1285
						51	0.1176
						52	0.1296
						53	0.1176
						54	0.1286
						55	0.3027
						56	0.3029
						57	0.1285
						58	0.1176
						59	0.1296
						60	0.1176
						61	0.1286
						62	0.3027
						63	0.3029
						64	0.1285
						65	0.1176
						66	0.1296
						67	0.1176
						68	0.1286
						69	0.3027
						70	0.4412
						71	0.2090
						72	0.1808
						73	0.1908
						74	0.1830
						75	0.2112
						76	0.4390
Average	0.3667	Average	0.5417	Average	0.7222	Average	0.1711

Power Evaluation for Full Models of Stage-Wise iDoE and DoE

	Term	Standard Error	VIF	R_i^2	Power SN 3	Power SN 10	Power SN 20
iDoE Stage 1	A - Time	8.37	116.79	0.99	0.0500	0.0877	0.2056
	B - DO	2.35	96.86	0.99	0.0900	0.5229	0.9806
	C - Temperature	2.35	96.86	0.99	0.0900	0.5229	0.9806
	AB	7.67	539.55	1	0.0500	0.0950	0.2359
	AC	7.67	539.55	1	0.0500	0.0950	0.2359
	BC	ALIASED					
	A^2	6.21	116.79	0.99	0.0700	0.3335	0.8630
	B^2	0.52	1.15	0.13	1.0000	1.0000	1.0000
	C^2	0.52	1.15	0.13	1.0000	1.0000	1.0000
	ABC	ALIASED					
	A^2B	5.69	223.5	1	0.0600	0.1328	0.3853
	A^2C	5.69	223.5	1	0.0600	0.1328	0.3853
iDoE Stage 2	A - Time	1.8	2.7	0.63	0.1200	0.7157	0.9989
	B - DO	0.46	2.87	0.65	0.8400	1.0000	1.0000
	C - Temperature	0.84	7	0.86	0.3700	0.9997	1.0000
	AB	2.2	3.15	0.68	0.1000	0.5432	0.9843
	AC	4.24	7.5	0.87	0.0600	0.1900	0.5750
	BC	0.87	6	0.83	0.3500	0.9994	1.0000
	A^2	8.05	2.7	0.63	0.0600	0.2059	0.6197
	B^2	0.46	1.13	0.11	1.0000	1.0000	1.0000
	C^2	0.61	1.63	0.38	0.9900	1.0000	1.0000
	ABC	4.24	6	0.83	0.0600	0.1900	0.5750
	A^2B	9.86	4.95	0.8	0.0500	0.0750	0.1530
	A^2C	11.38	4.5	0.78	0.0500	0.0687	0.1267
iDoE Stage 3	A - Time	14.8	170.43	0.99	0.0500	0.0600	0.0900
	B - DO	7.82	519.32	1	0.0500	0.0800	0.1800
	C - Temperature	6.74	454.07	1	0.0500	0.0900	0.2300
	AB	20.58	2042.88	1	0.0500	0.0500	0.0700
	AC	18.83	2009.7	1	0.0500	0.0600	0.0700
	BC	3.47	72.45	0.99	0.0600	0.2200	0.6400
	A^2	9.68	170.27	0.99	0.0600	0.1400	0.3900
	B^2	1.78	14.25	0.93	0.2800	0.9900	1.0000
	C^2	1.44	6.74	0.85	0.3900	1.0000	1.0000
	ABC	3.63	44.76	0.98	0.0600	0.2000	0.6000
	A^2B	13.36	645.94	1	0.0500	0.0600	0.0900
	A^2C	12.31	644.59	1	0.0500	0.0600	0.1000
DoE Growth Phase	A - Time	0.17	1.35	0.26	1.0000	1.0000	1.0000
	B - DO	0.2	2.33	0.57	1.0000	1.0000	1.0000
	C - Temperature	0.22	2.37	0.58	1.0000	1.0000	1.0000
	AB	0.2	1.51	0.34	1.0000	1.0000	1.0000
	AC	0.2	1.43	0.3	1.0000	1.0000	1.0000
	BC	0.17	1.16	0.14	1.0000	1.0000	1.0000
	A^2	0.21	1.35	0.26	1.0000	1.0000	1.0000
	B^2	0.28	1.18	0.15	1.0000	1.0000	1.0000
	C^2	0.28	1.09	0.08	1.0000	1.0000	1.0000
	ABC	0.2	1.16	0.14	1.0000	1.0000	1.0000
	A^2B	0.24	2.84	0.65	1.0000	1.0000	1.0000
	A^2C	0.25	2.91	0.66	1.0000	1.0000	1.0000

Abbreviations: A = Time, B = Dissolved Oxygen, C = Temperature, VIF = variable inflation factor, SN = signal-to-noise

iDoE Stage 1													
	Intercept	A - Time	B - DO	C - Temperature	AB	AC	BC	A^2	B^2	C^2	ABC	A^2B	A^2C
Intercept	1.E+00	9.E-01	6.E-01	6.E-01	6.E-01	6.E-01		9.E-01	-2.E-01	-2.E-01		6.E-01	6.E-01
A - Time	9.E-01	1.E+00	6.E-01	6.E-01	7.E-01	7.E-01		1.E+00	2.E-16	-9.E-16		6.E-01	6.E-01
B - DO	6.E-01	6.E-01	1.E+00	4.E-01	1.E+00	4.E-01		6.E-01	2.E-02	-2.E-02		9.E-01	4.E-01
C - Temperature	6.E-01	6.E-01	4.E-01	1.E+00	4.E-01	1.E+00		6.E-01	-2.E-02	2.E-02		4.E-01	9.E-01
AB	6.E-01	7.E-01	1.E+00	4.E-01	1.E+00	4.E-01		6.E-01	2.E-16	-6.E-16		1.E+00	4.E-01
AC	6.E-01	7.E-01	4.E-01	1.E+00	4.E-01	1.E+00		6.E-01	-5.E-16	-1.E-15		4.E-01	1.E+00
BC	aliased						aliased				aliased		
A^2	9.E-01	1.E+00	6.E-01	6.E-01	6.E-01	6.E-01		1.E+00	2.E-16	-9.E-16		7.E-01	7.E-01
B^2	-2.E-01	2.E-16	2.E-02	-2.E-02	2.E-16	-5.E-16		2.E-16	1.E+00	9.E-02		2.E-16	-5.E-16
C^2	-2.E-01	-9.E-16	-2.E-02	2.E-02	-6.E-16	-1.E-15		-9.E-16	9.E-02	1.E+00		-6.E-16	-1.E-15
ABC	aliased												
A^2B	6.E-01	6.E-01	9.E-01	4.E-01	1.E+00	4.E-01		7.E-01	2.E-16	-6.E-16		1.E+00	4.E-01
A^2C	6.E-01	6.E-01	4.E-01	9.E-01	4.E-01	1.E+00		7.E-01	-5.E-16	-1.E-15		4.E-01	1.E+00

iDoE Stage 2													
	Intercept	A - Time	B - DO	C - Temperature	AB	AC	BC	A^2	B^2	C^2	ABC	A^2B	A^2C
Intercept	1.E+00	2.E-01	6.E-03	3.E-18	-9.E-02	-2.E-17	-5.E-17	-5.E-01	-6.E-01	-3.E-01	3.E-17	2.E-01	1.E-16
A - Time	2.E-01	1.E+00	-1.E-01	-1.E-16	-4.E-01	6.E-17	1.E-16	-7.E-01	1.E-16	-1.E-16	-9.E-17	3.E-01	7.E-17
B - DO	6.E-03	-1.E-01	1.E+00	-2.E-17	3.E-01	-7.E-17	-2.E-17	2.E-01	-1.E-01	-4.E-01	2.E-17	-6.E-01	1.E-16
C - Temperature	3.E-18	-1.E-16	-2.E-17	1.E+00	9.E-17	-2.E-01	-8.E-01	7.E-17	-3.E-17	-9.E-19	3.E-01	3.E-19	-4.E-01
AB	-9.E-02	-4.E-01	3.E-01	9.E-17	1.E+00	-1.E-16	-1.E-16	3.E-01	3.E-16	-6.E-17	1.E-16	-7.E-01	-1.E-17
AC	-2.E-17	6.E-17	-7.E-17	-2.E-01	-1.E-16	1.E+00	3.E-01	-2.E-17	7.E-17	-4.E-18	-8.E-01	4.E-17	-4.E-01
BC	-5.E-17	1.E-16	-2.E-17	-8.E-01	-1.E-16	3.E-01	1.E+00	-6.E-17	1.E-16	-4.E-18	-4.E-01	2.E-17	4.E-17
A^2	-5.E-01	-7.E-01	2.E-01	7.E-17	3.E-01	-2.E-17	-6.E-17	1.E+00	-2.E-16	2.E-16	3.E-17	-4.E-01	-6.E-17
B^2	-6.E-01	1.E-16	-1.E-01	-3.E-17	3.E-16	7.E-17	1.E-16	-2.E-16	1.E+00	-1.E-01	-5.E-17	-1.E-16	-2.E-16
C^2	-3.E-01	-1.E-16	-4.E-01	-9.E-19	-6.E-17	-4.E-18	-4.E-18	2.E-16	-1.E-01	1.E+00	-1.E-17	-3.E-17	2.E-33
ABC	3.E-17	-9.E-17	2.E-17	3.E-01	1.E-16	-8.E-01	-4.E-01	3.E-17	-5.E-17	-1.E-17	1.E+00	2.E-33	-4.E-17
A^2B	2.E-01	3.E-01	-6.E-01	3.E-19	-7.E-01	4.E-17	2.E-17	-4.E-01	-1.E-16	-3.E-17	2.E-33	1.E+00	-4.E-17
A^2C	1.E-16	7.E-17	1.E-16	-4.E-01	-1.E-17	-4.E-01	4.E-17	-6.E-17	-2.E-16	2.E-33	-4.E-17	-4.E-17	1.E+00

iDoE Stage 3													
	Intercept	A - Time	B - DO	C - Temperature	AB	AC	BC	A^2	B^2	C^2	ABC	A^2B	A^2C
Intercept	1.E+00	-1.E+00	-3.E-01	-2.E-01	2.E-01	2.E-01	1.E-01	1.E+00	6.E-02	-1.E-01	-6.E-02	-2.E-01	-2.E-01
A - Time	-1.E+00	1.E+00	2.E-01	2.E-01	-2.E-01	-2.E-01	-2.E-02	-1.E+00	2.E-16	-7.E-16	3.E-02	2.E-01	2.E-01
B - DO	-3.E-01	2.E-01	1.E+00	8.E-02	-9.E-01	-5.E-02	-4.E-01	-2.E-01	-3.E-01	3.E-01	2.E-01	9.E-01	5.E-02
C - Temperature	-2.E-01	2.E-01	8.E-02	1.E+00	-5.E-02	-1.E+00	-8.E-02	-2.E-01	-1.E-01	9.E-02	1.E-02	5.E-02	1.E+00
AB	2.E-01	-2.E-01	-9.E-01	-5.E-02	1.E+00	5.E-02	9.E-02	2.E-01	9.E-16	-4.E-16	-1.E-01	-1.E+00	-5.E-02
AC	2.E-01	-2.E-01	-5.E-02	-1.E+00	5.E-02	1.E+00	5.E-03	2.E-01	1.E-15	-1.E-15	-7.E-03	-5.E-02	-1.E+00
BC	1.E-01	-2.E-02	-4.E-01	-8.E-02	9.E-02	5.E-03	1.E+00	7.E-16	6.E-01	-6.E-01	-8.E-01	7.E-16	7.E-16
A^2	1.E+00	-1.E+00	-2.E-01	-2.E-01	2.E-01	2.E-01	7.E-16	1.E+00	-2.E-16	8.E-16	-9.E-16	-2.E-01	-2.E-01
B^2	6.E-02	2.E-16	-3.E-01	-1.E-01	9.E-16	1.E-15	6.E-01	-2.E-16	1.E+00	-9.E-01	4.E-16	-1.E-15	-1.E-15
C^2	-1.E-01	-7.E-16	3.E-01	9.E-02	-4.E-16	-1.E-15	-6.E-01	8.E-16	-9.E-01	1.E+00	-3.E-16	4.E-16	1.E-15
ABC	-6.E-02	3.E-02	2.E-01	1.E-02	-1.E-01	-7.E-03	-8.E-01	-9.E-16	4.E-16	-3.E-16	1.E+00	-1.E-15	-2.E-15
A^2B	-2.E-01	2.E-01	9.E-01	5.E-02	-1.E+00	-5.E-02	7.E-16	-2.E-01	-1.E-15	4.E-16	-1.E-15	1.E+00	5.E-02
A^2C	-2.E-01	2.E-01	5.E-02	1.E+00	-5.E-02	-1.E+00	7.E-16	-2.E-01	-1.E-15	1.E-15	-2.E-15	5.E-02	1.E+00

DoE Growth Phase													
	Intercept	A - Time	B - DO	C - Temperature	AB	AC	BC	A^2	B^2	C^2	ABC	A^2B	A^2C
**Intercept**	1.E+00	-1.E-01	-4.E-17	5.E-02	-6.E-17	1.E-02	6.E-17	-4.E-01	-5.E-01	-4.E-01	4.E-19	3.E-18	7.E-02
**A - Time**	-1.E-01	1.E+00	3.E-17	2.E-02	-9.E-18	-1.E-01	-5.E-17	5.E-01	3.E-17	5.E-17	-3.E-17	-2.E-17	-5.E-02
**B - DO**	-4.E-17	3.E-17	1.E+00	1.E-17	-2.E-01	3.E-18	-2.E-01	4.E-17	3.E-17	2.E-17	-5.E-02	-7.E-01	-2.E-17
**C - Temperature**	5.E-02	2.E-02	1.E-17	1.E+00	4.E-17	-2.E-01	-2.E-17	1.E-01	-2.E-01	-3.E-02	2.E-19	2.E-17	-7.E-01
**AB**	-6.E-17	-9.E-18	-2.E-01	4.E-17	1.E+00	8.E-18	-6.E-02	2.E-17	8.E-17	-2.E-17	-3.E-01	5.E-01	-5.E-17
**AC**	1.E-02	-1.E-01	3.E-18	-2.E-01	8.E-18	1.E+00	3.E-17	-5.E-02	3.E-17	-4.E-17	2.E-17	-1.E-17	5.E-01
**BC**	6.E-17	-5.E-17	-2.E-01	-2.E-17	-6.E-02	3.E-17	1.E+00	-7.E-17	-3.E-17	-3.E-18	2.E-01	-3.E-17	5.E-17
**A^2**	-4.E-01	5.E-01	4.E-17	1.E-01	2.E-17	-5.E-02	-7.E-17	1.E+00	2.E-02	-2.E-02	-6.E-18	-2.E-17	-1.E-01
**B^2**	-5.E-01	3.E-17	3.E-17	-2.E-01	8.E-17	3.E-17	-3.E-17	2.E-02	1.E+00	-3.E-01	-4.E-18	5.E-19	-3.E-02
**C^2**	-4.E-01	5.E-17	2.E-17	-3.E-02	-2.E-17	-4.E-17	-3.E-18	-2.E-02	-3.E-01	1.E+00	7.E-18	-1.E-17	2.E-02
**ABC**	4.E-19	-3.E-17	-5.E-02	2.E-19	-3.E-01	2.E-17	2.E-01	-6.E-18	-4.E-18	7.E-18	1.E+00	-2.E-17	4.E-18
**A^2B**	3.E-18	-2.E-17	-7.E-01	2.E-17	5.E-01	-1.E-17	-3.E-17	-2.E-17	5.E-19	-1.E-17	-2.E-17	1.E+00	-4.E-18
**A^2C**	7.E-02	-5.E-02	-2.E-17	-7.E-01	-5.E-17	5.E-01	5.E-17	-1.E-01	-3.E-02	2.E-02	4.E-18	-4.E-18	1.E+00

Aliasing of Model Terms

	Estimated Term	Aliased Terms
iDoE Stage 1	Intercept	= Intercept - BC + 0.556 * AB^2 + 0.556 * AC^2
	A - Time	= A - ABC + 0.833 * AB^2 + 0.833 * AC^2
	B - DO	= B - BC - 0.0667 * AB^2 + 0.0667 * AC^2
	C - Temperature	= C - BC + 0.0667 * AB^2 - 0.0667 * AC^2
	AB	= AB - ABC - 0.1 * AB^2 + 0.1 * AC^2
	AC	= AC - ABC + 0.1 * AB^2 - 0.1 * AC^2
	A^2	= A^2
	B^2	= B^2 - 0.667 * AB^2
	C^2	= C^2 - 0.667 * AC^2
	A^2B	= A^2B
	A^2C	= A^2C
iDoE Stage 2	Intercept	= Intercept - 0.05 * AB^2 - 0.025 * AC^2
	A - Time	= A + 0.6 * AB^2 + 0.3 * AC^2
	B - DO	= B - 0.0167 * AB^2 - 0.0292 * AC^2
	C - Temperature	= C
	AB	= AB + 0.2 * AB^2 + 0.35 * AC^2
	AC	= AC
	BC	= BC
	A^2	= A^2
	B^2	= B^2 + 0.0833 * AB^2
	C^2	= C^2 + 0.0833 * AC^2
	ABC	= ABC
	A^2B	= A^2B
	A^2C	= A^2C
iDoE Stage 3	Intercept	= Intercept - 0.219 * AB^2 - 0.492 * AC^2
	A - Time	= A + 0.303 * AB^2 + 0.682 * AC^2
	B - DO	= B - 0.503 * AB^2 + 0.492 * AC^2
	C - Temperature	= C - 0.35 * AB^2 - 0.0657 * AC^2
	AB	= AB + 0.697 * AB^2 - 0.682 * AC^2
	AC	= AC + 0.485 * AB^2 + 0.0909 * AC^2
	BC	= BC + 0.503 * AB^2 - 0.492 * AC^2
	A^2	= A^2
	B^2	= B^2 + 0.722 * AB^2
	C^2	= C^2 + 0.722 * AC^2
	ABC	= ABC - 0.697 * AB^2 + 0.682 * AC^2
	A^2B	= A^2B
	A^2C	= A^2C
DoE Growth Phase	Intercept	= Intercept + 0.152 * AB^2 + 0.129 * AC^2
	A - Time	= A + 0.712 * AB^2 + 0.678 * AC^2
	B - DO	= B
	C - Temperature	= C + 0.0254 * AB^2 + 0.0146 * AC^2
	AB	= AB
	AC	= AC + 0.172 * AB^2 + 0.0429 * AC^2
	BC	= BC
	A^2	= A^2 - 0.00566 * AB^2 + 0.00383 * AC^2
	B^2	= B^2 - 0.212 * AB^2 + 0.00803 * AC^2
	C^2	= C^2 + 0.00927 * AB^2 - 0.206 * AC^2
	ABC	= ABC
	A^2B	= A^2B
	A^2C	= A^2C + 0.00799 * AB^2 - 0.0054 * AC^2
