# Supplementary material for: Applying intensified design of experiments to mammalian cell culture processes
Source: Eng Life Sci. 2021 Nov 24;22(12):784–95. doi: 10.1002/elsc.202100123 (PMC9731596; doi:10.1002/elsc.202100123)
Supplement: Supplementary file 2 — Supporting information. [file ELSC-22-784-s003.pdf]

Diagnostic Plots Intensified Design of Experiments

Stage 1

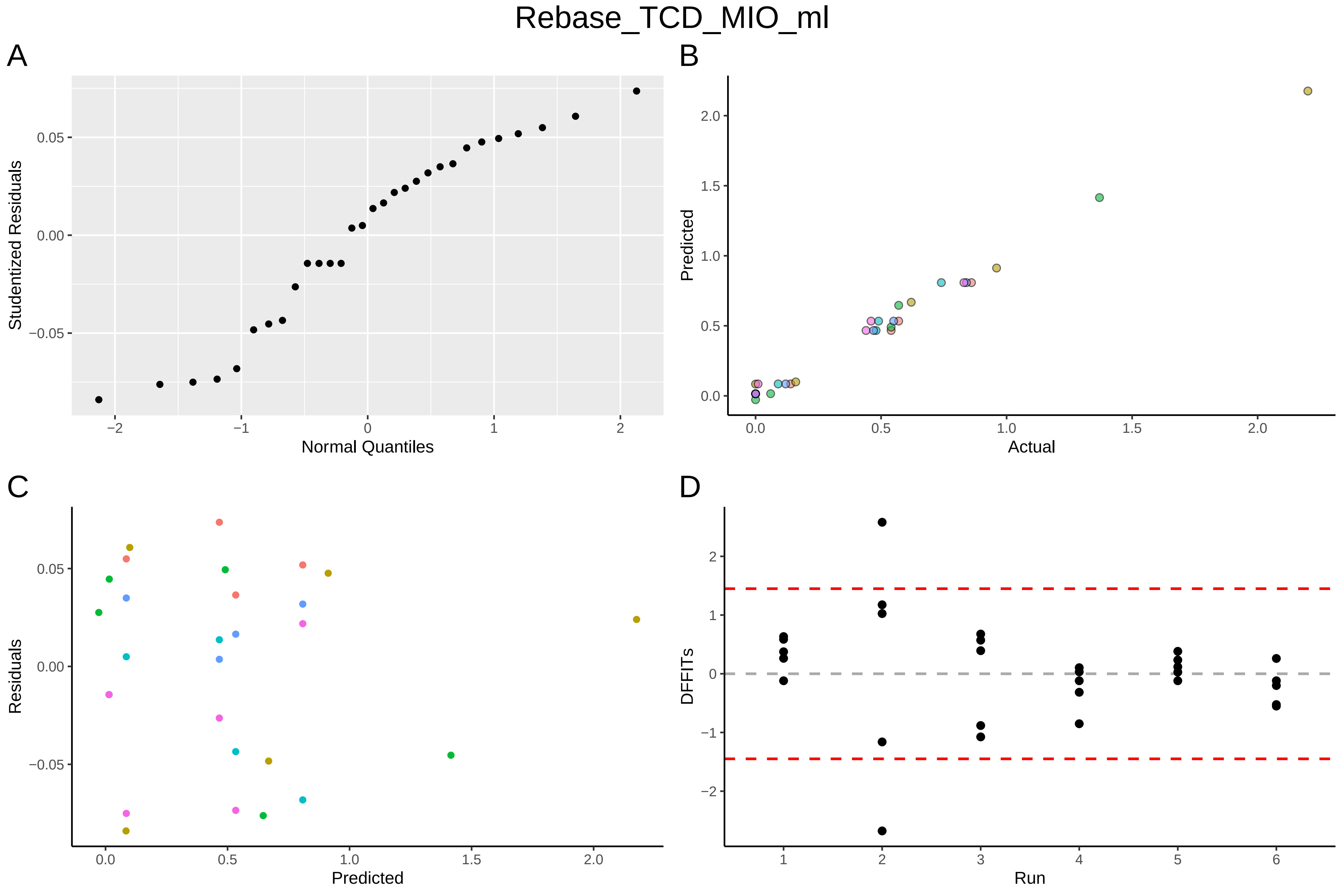

Rebase_TCD_MIO_ml
A
Studentized Residuals
Normal Quantiles
0.05
0.00
−0.05
−2
−1
0
1
2
B
Predicted
Actual
0.0
0.5
1.0
1.5
2.0
C
Residuals
Predicted
D
DFFITs
Run
1
2
3
4
5
6

Rebase_VCD_MIO_ml

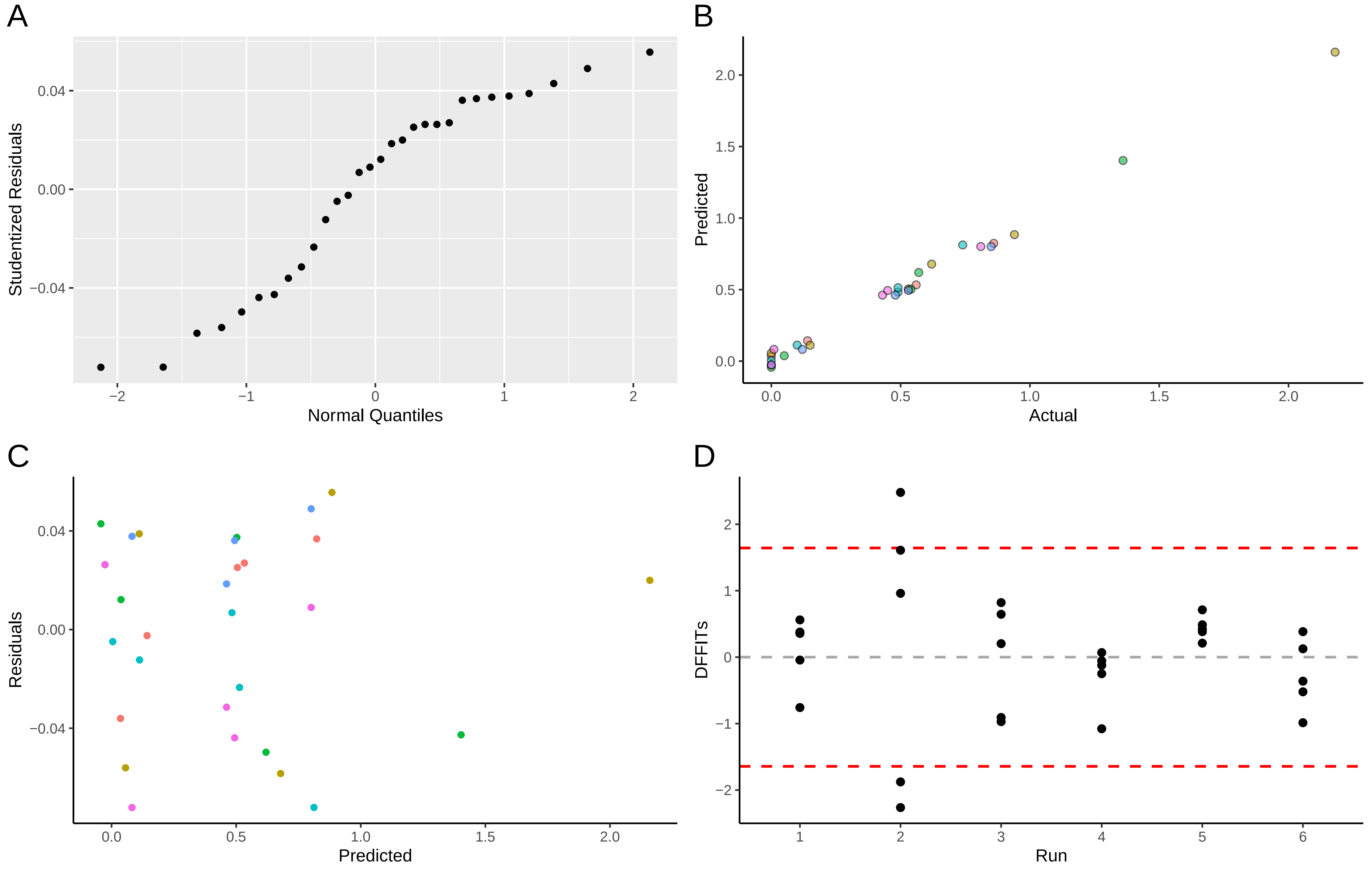

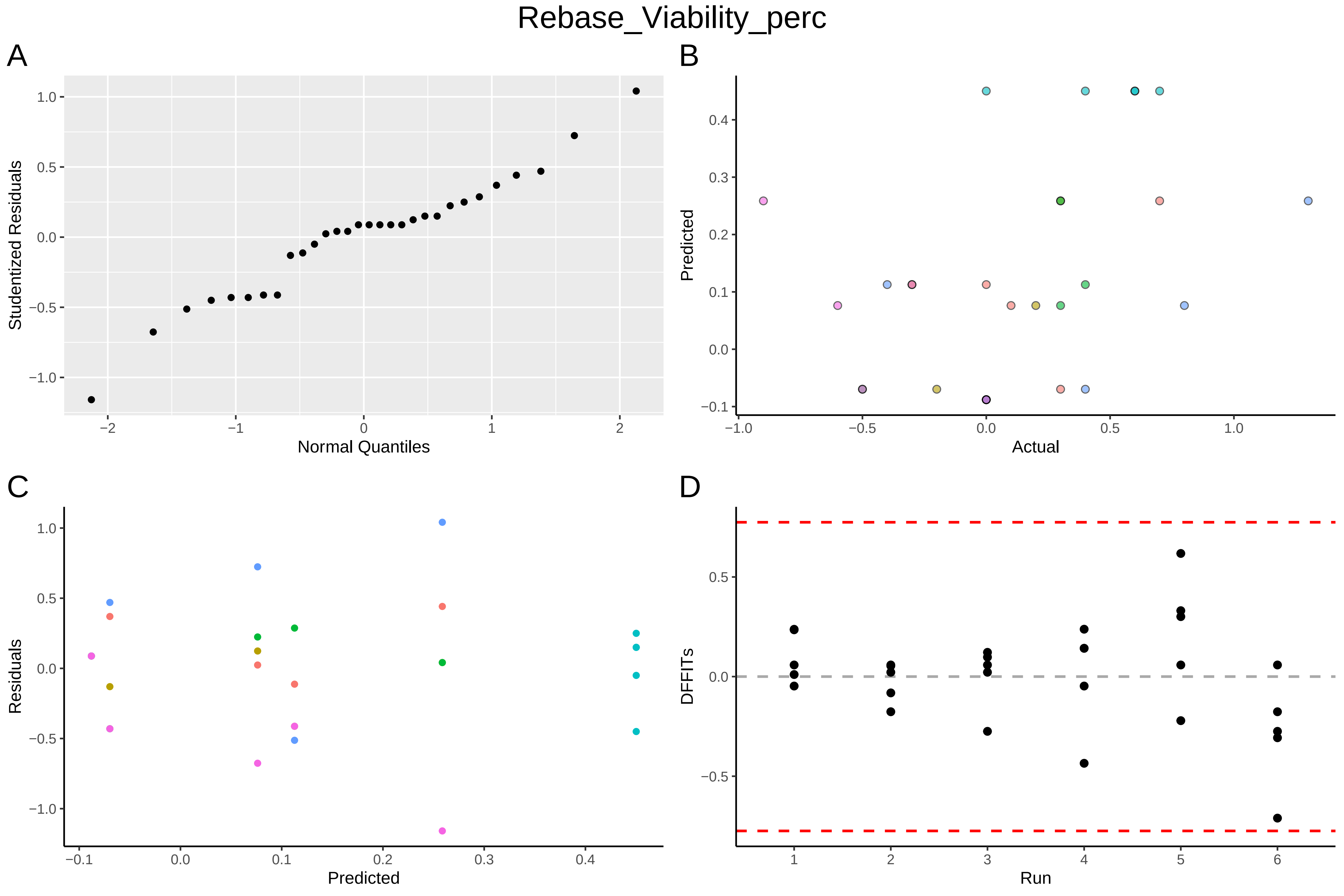

Rebase_Viability_perc
A
Studentized Residuals
Normal Quantiles
B
Predicted
Actual
C
Residuals
Predicted
D
DFFITs
Run

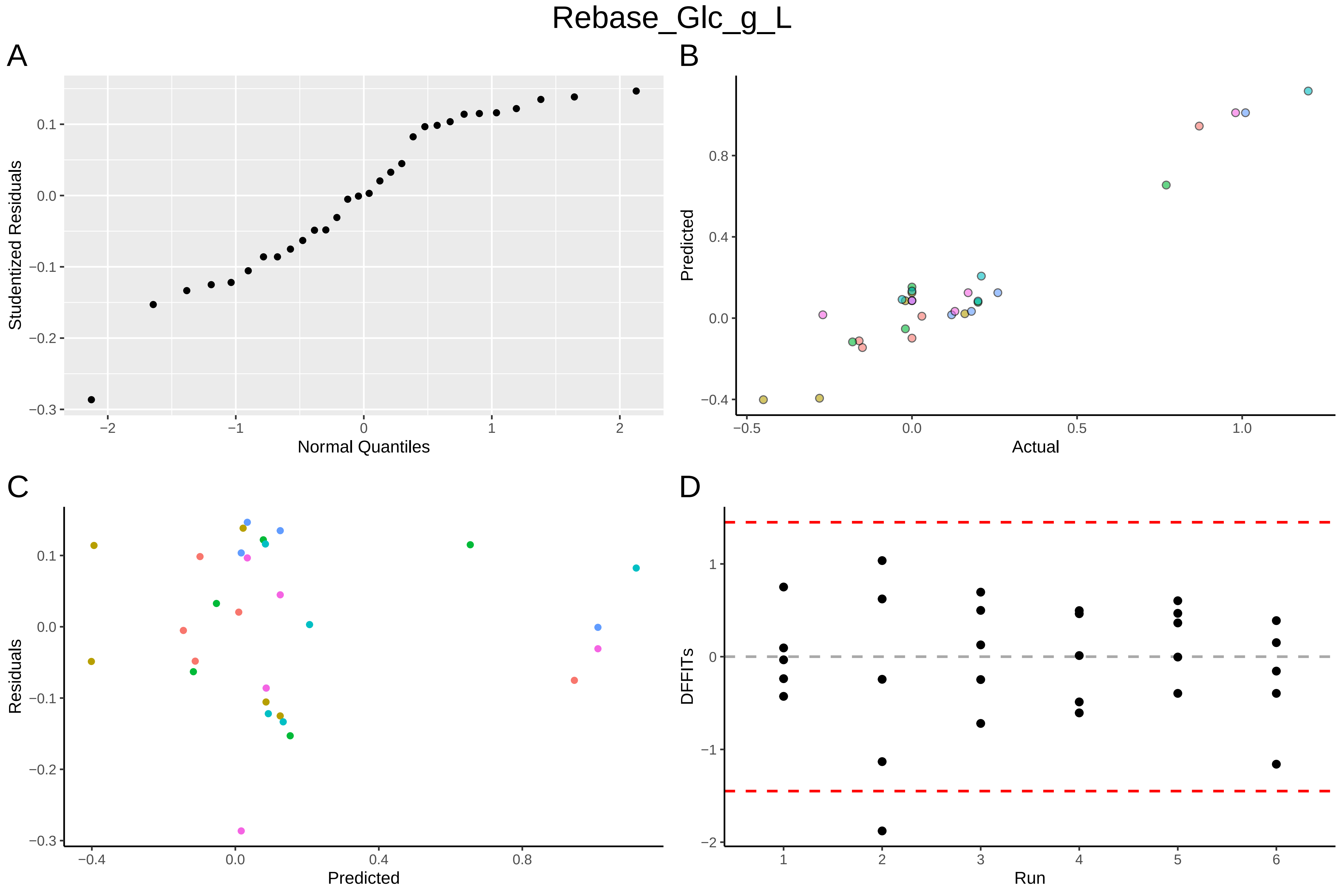

Rebase_Glc_g_L
A
Studentized Residuals
Normal Quantiles
B
Predicted
Actual
C
Residuals
Predicted
D
DFFITs
Run

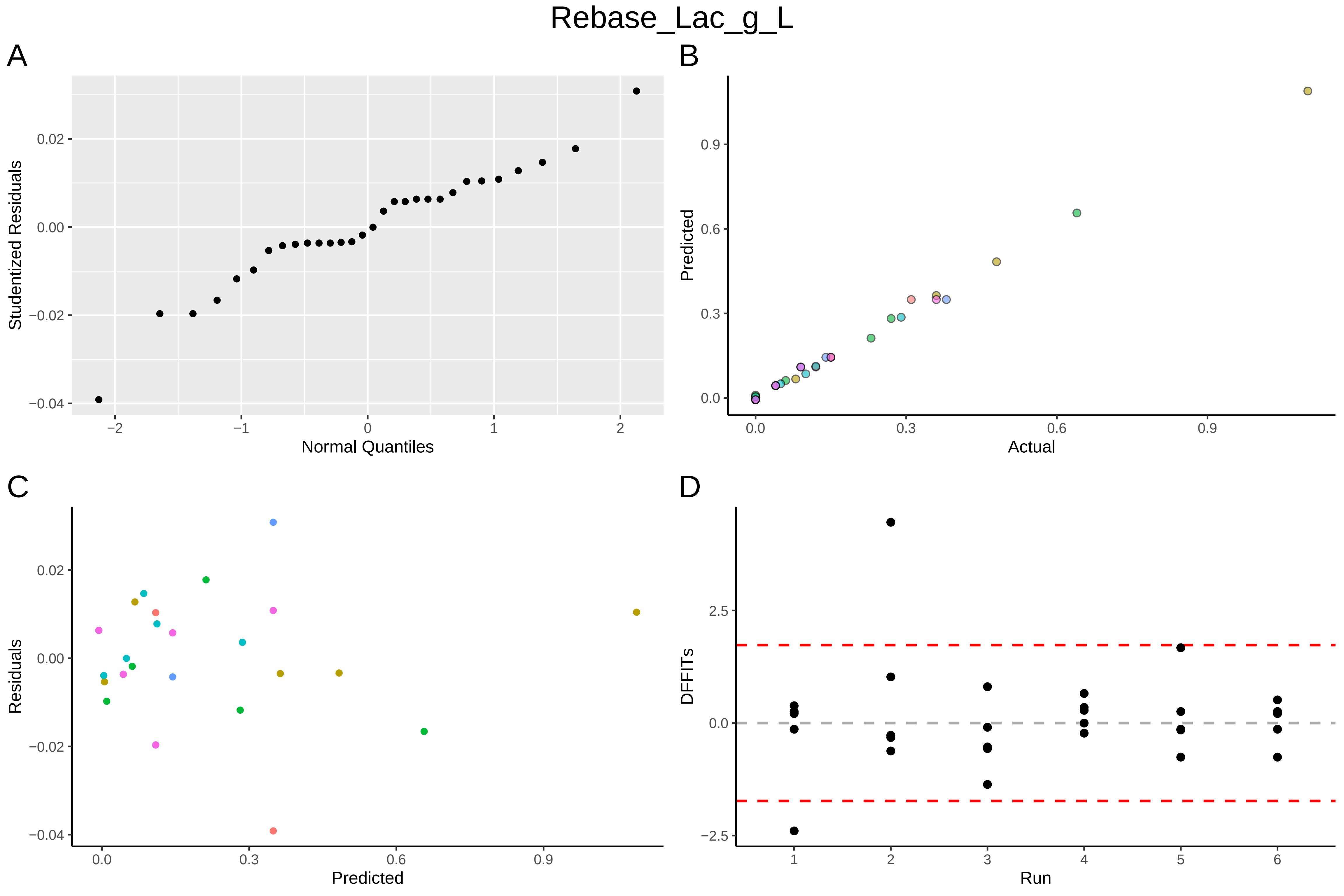
Rebase_Lac_g_L
A
Studentized Residuals
Normal Quantiles
0.02
0.00
−0.02
−0.04
−2
−1
0
1
2
B
Predicted
Actual
0.9
0.6
0.3
0.0
0.0
0.3
0.6
0.9
C
Residuals
Predicted
0.02
0.00
−0.02
−0.04
0.0
0.3
0.6
0.9
D
DFFITs
Run
2.5
0.0
−2.5
1
2
3
4
5
6

Diagnostic Plots Intensified Design of Experiments

Stage 2

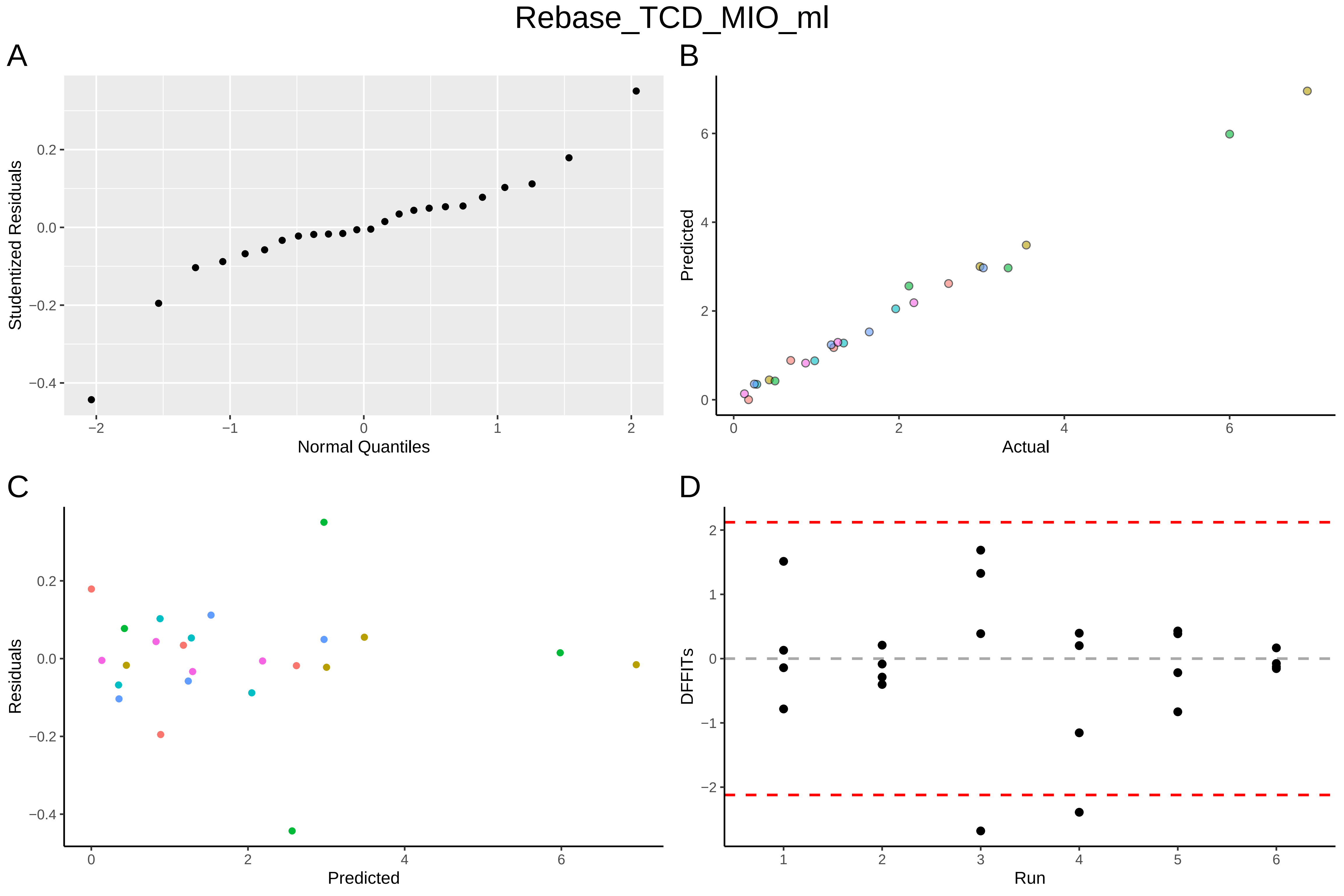

Rebase_TCD_MIO_ml
A
B
C
D
Studentized Residuals
Normal Quantiles
Predicted
Actual
Residuals
DFFITs
Run

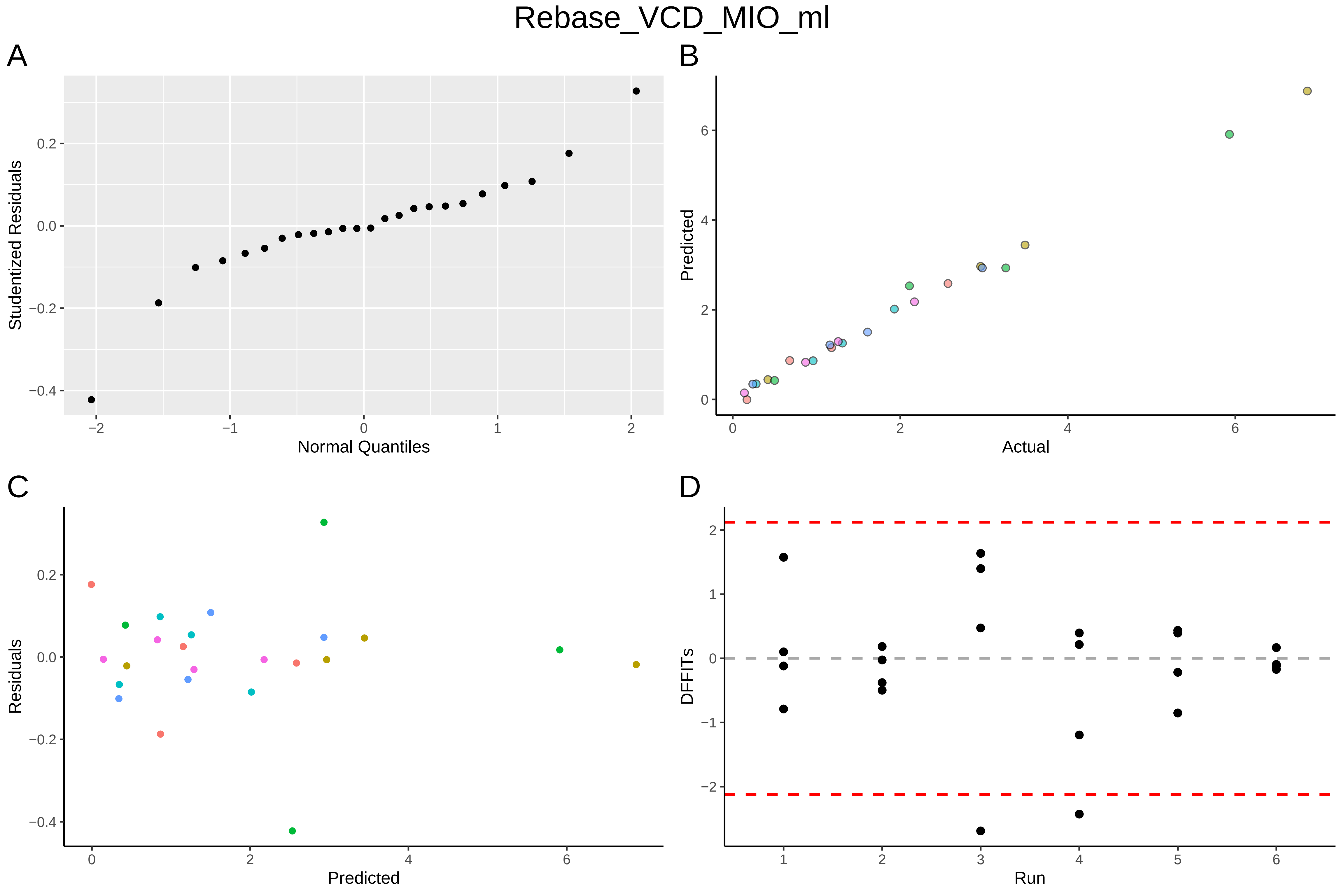
Rebase_VCD_MIO_ml
A
Studentized Residuals
Normal Quantiles
B
Predicted
Actual
C
Residuals
Predicted
D
DFFITs
Run

Rebase_Viability_perc

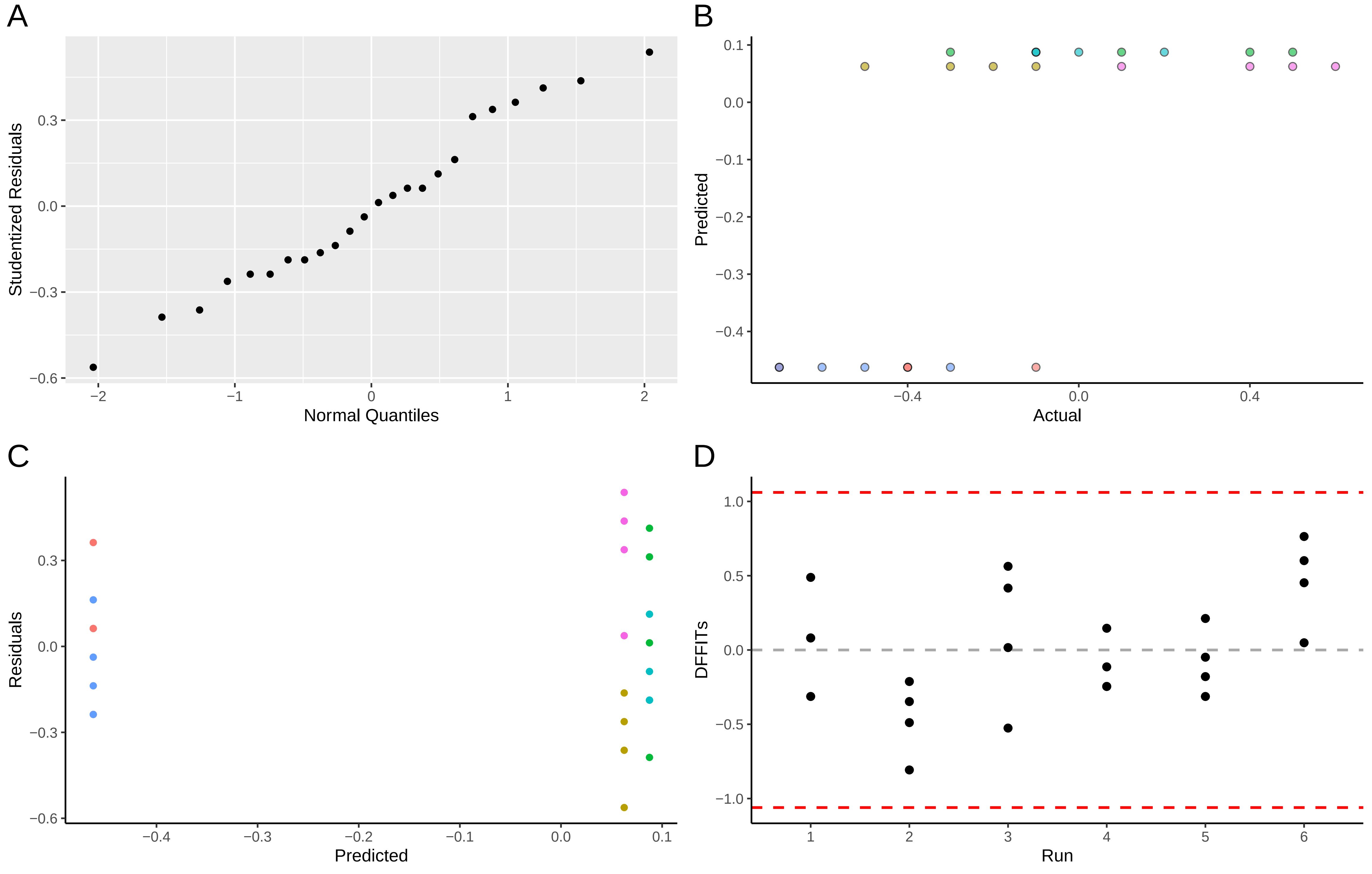

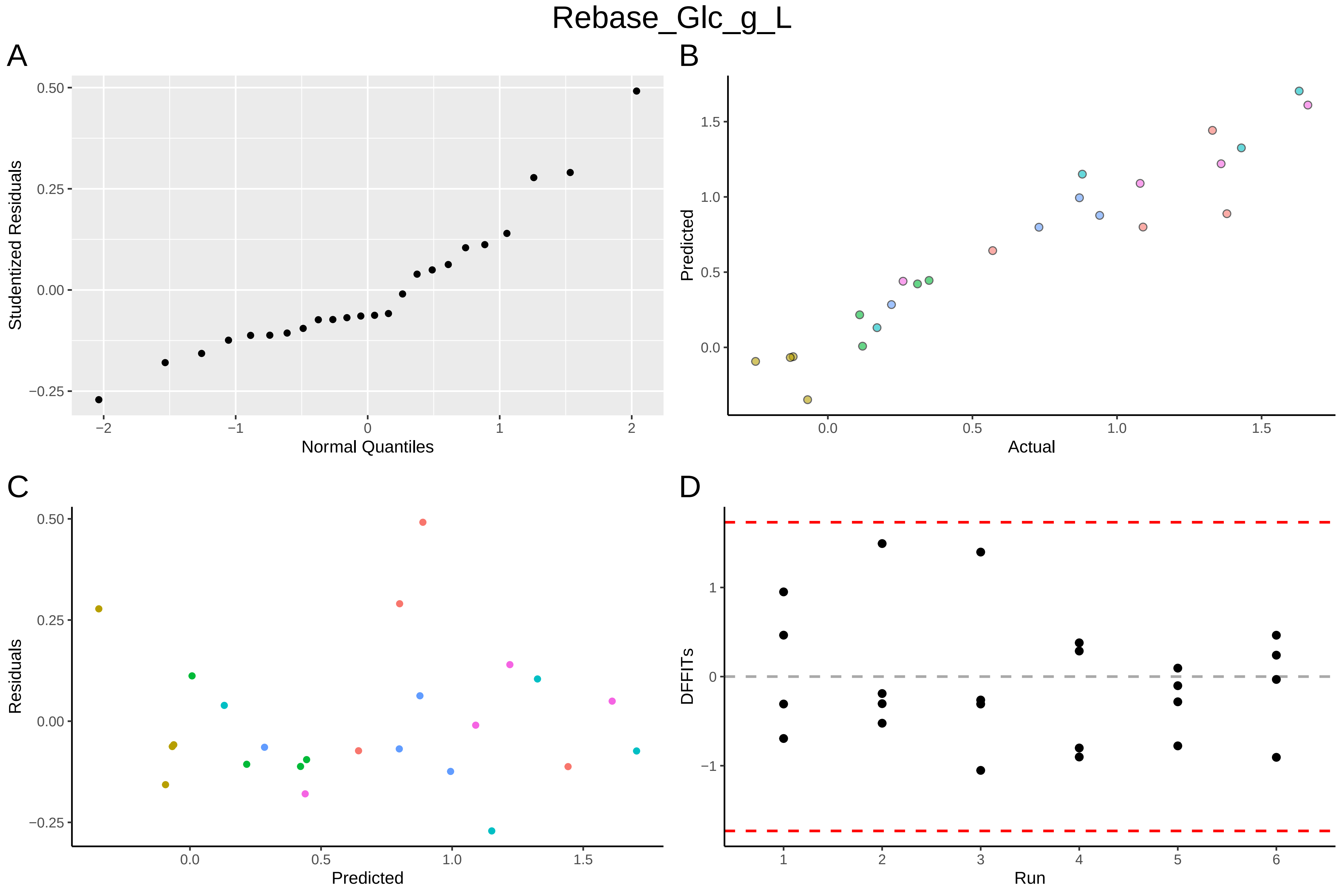

Rebase_Glc_g_L
A
Studentized Residuals
Normal Quantiles
B
Predicted
Actual
C
Residuals
Predicted
D
DFFITs
Run

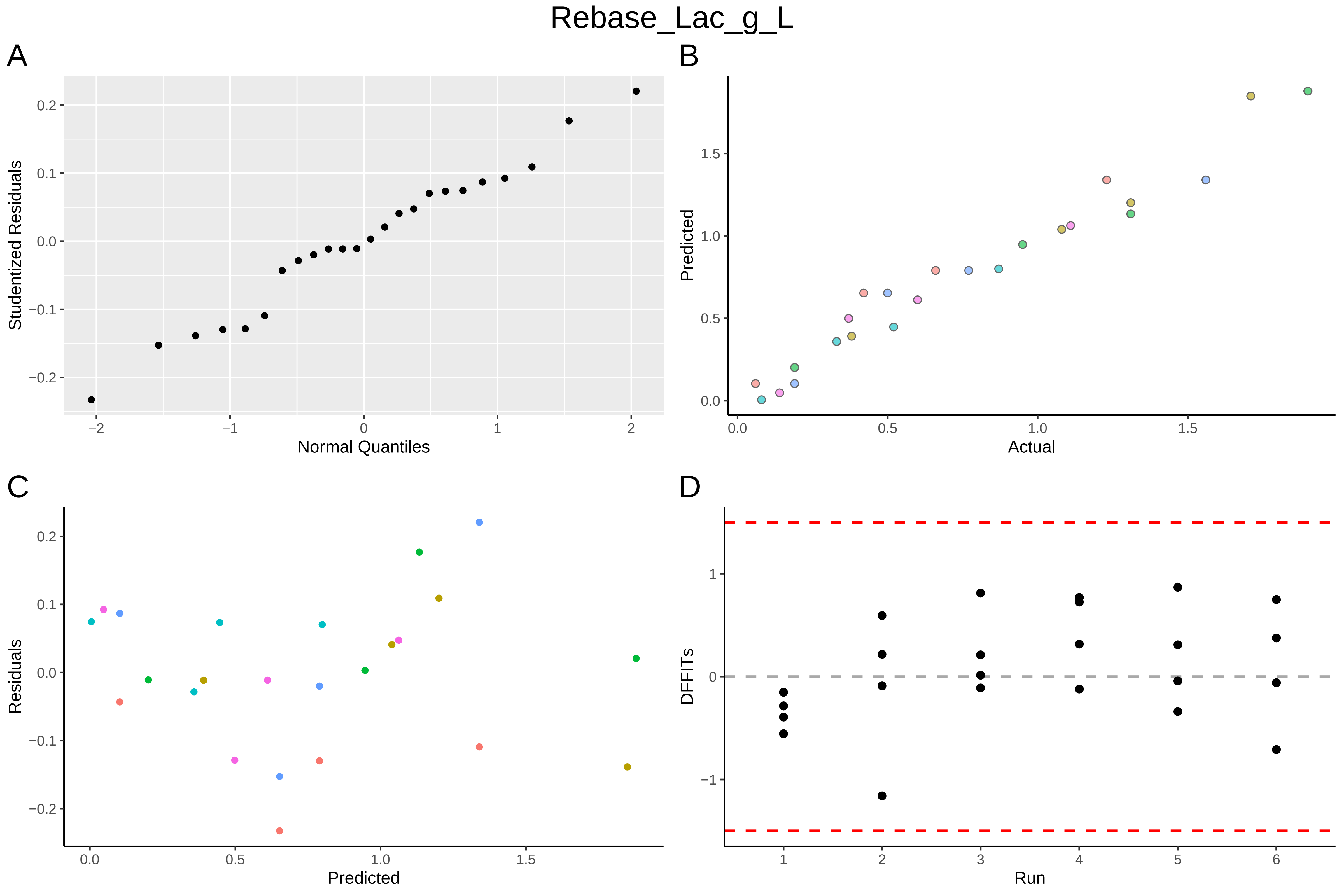

Rebase_Lac_g_L
A
Studentized Residuals
Normal Quantiles
B
Predicted
Actual
C
Residuals
Predicted
D
DFFITs
Run

Diagnostic Plots Intensified Design of Experiments

Stage 3

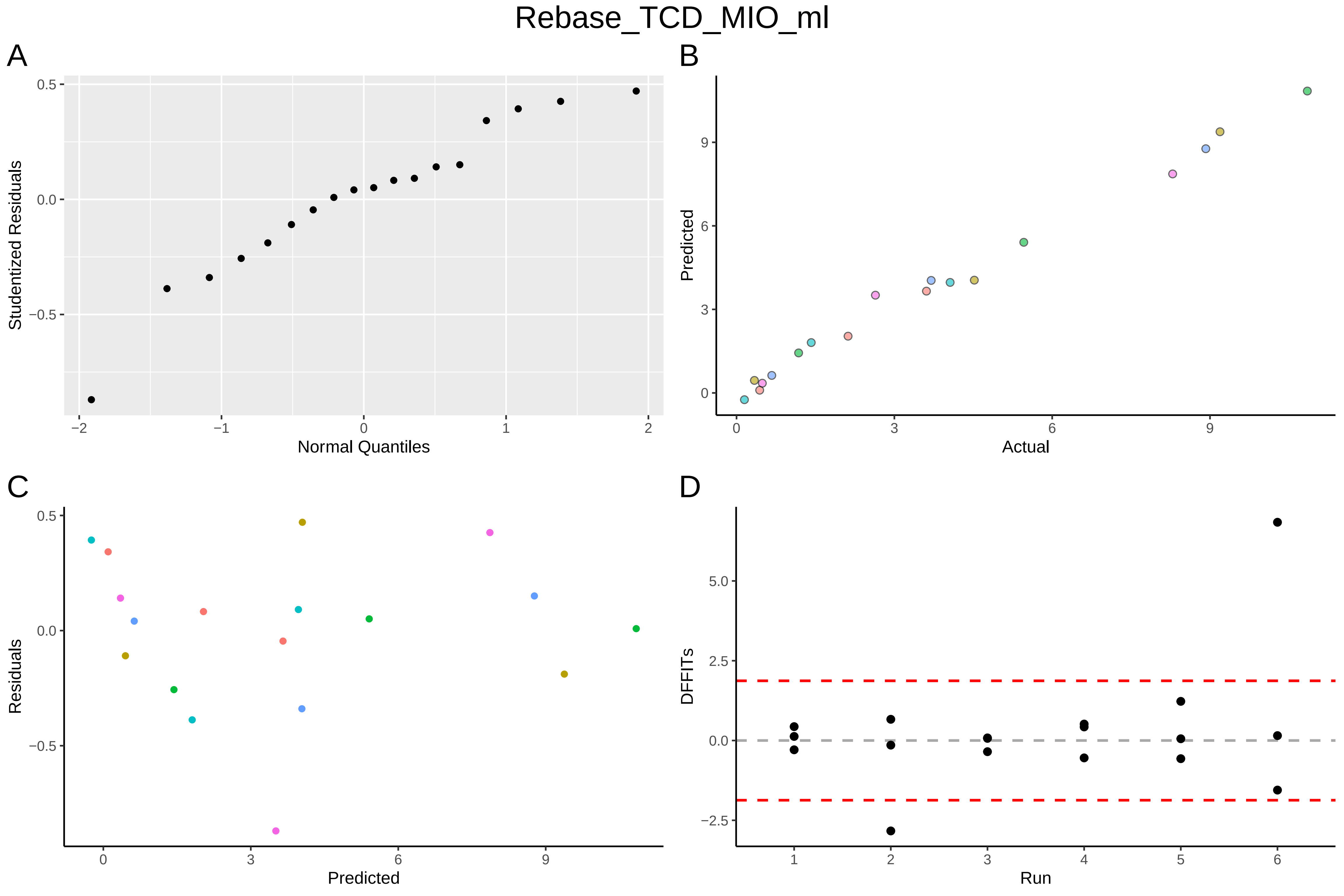
Rebase_TCD_MIO_ml
A
Studentized Residuals
Normal Quantiles
B
Predicted
Actual
C
Residuals
Predicted
D
DFFITs
Run

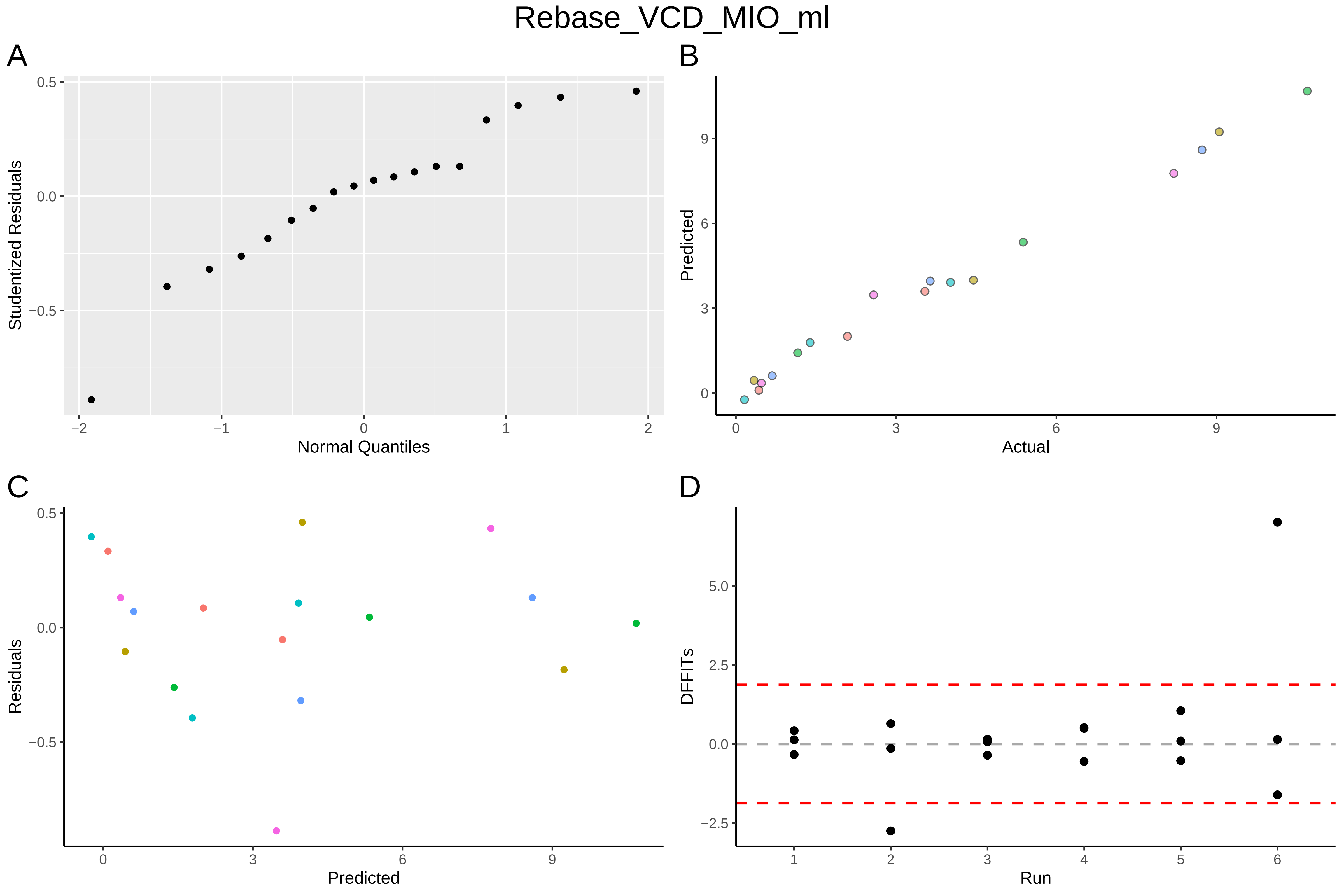

Rebase_VCD_MIO_ml
A
Studentized Residuals
Normal Quantiles
B
Predicted
Actual
C
Residuals
Predicted
D
DFFITs
Run

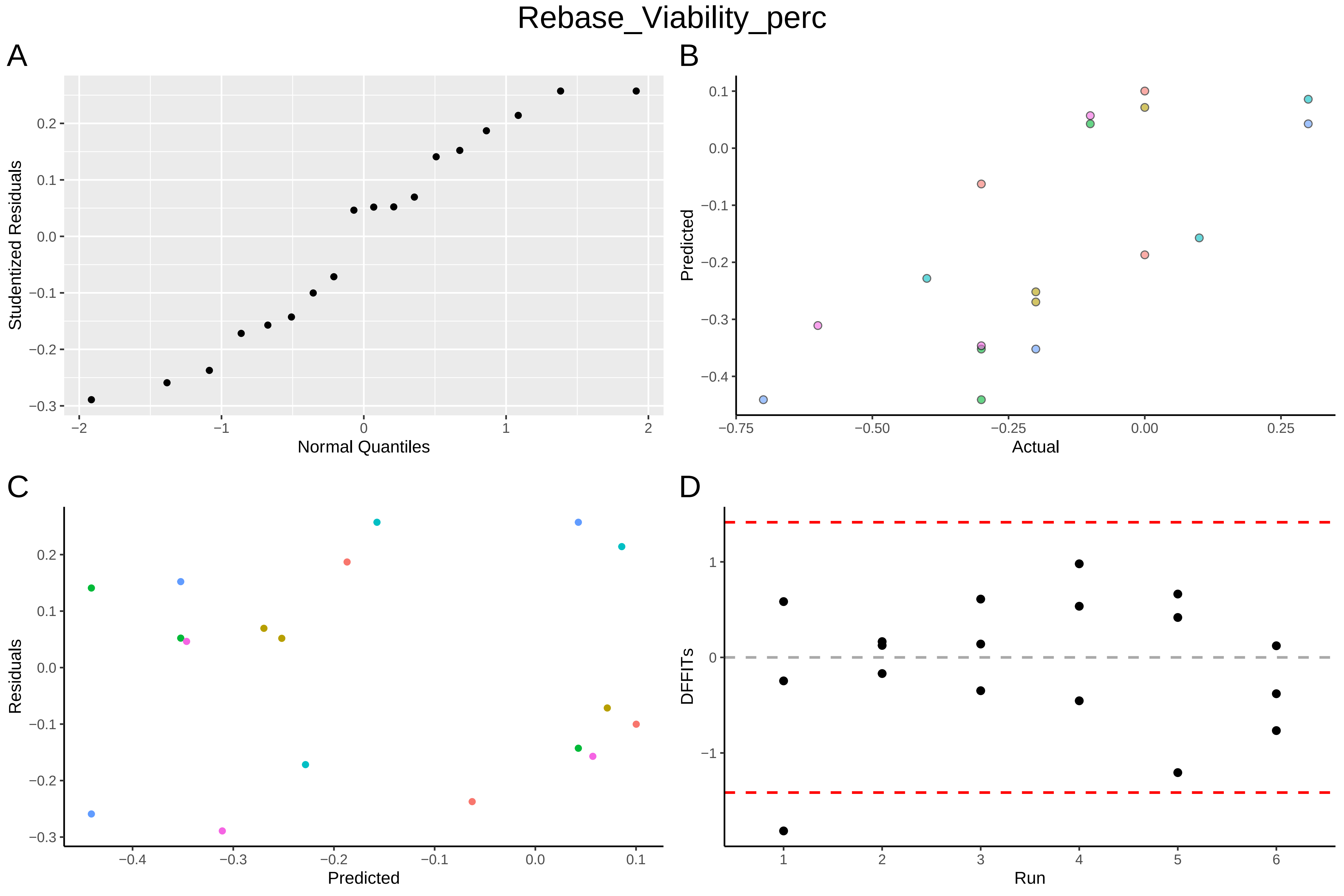
Rebase_Viability_perc
A
Studentized Residuals
Normal Quantiles
B
Predicted
Actual
C
Residuals
Predicted
D
DFFITs
Run

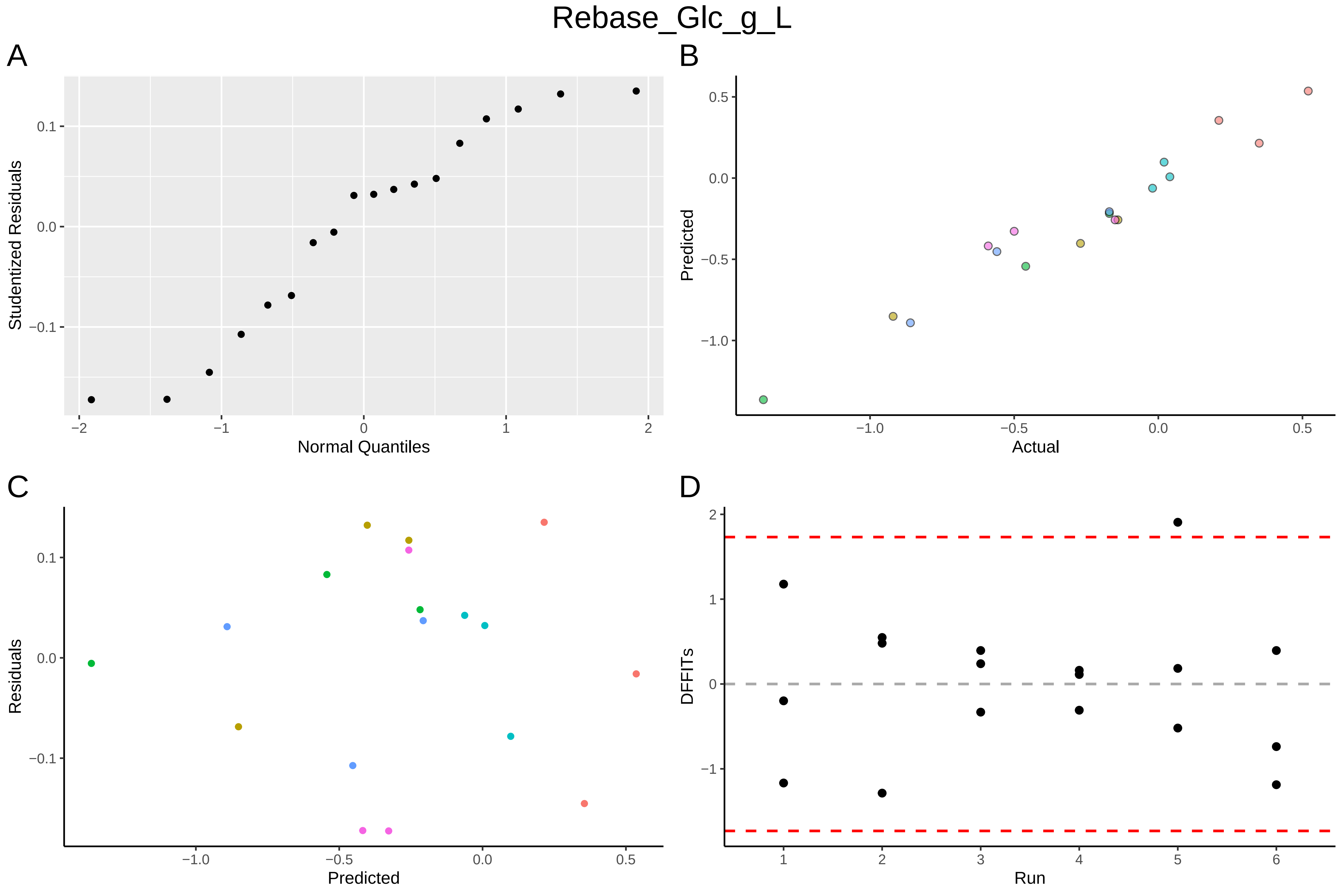
Rebase_Glc_g_L
A
Studentized Residuals
Normal Quantiles
B
Predicted
Actual
C
Residuals
Predicted
D
DFFITs
Run

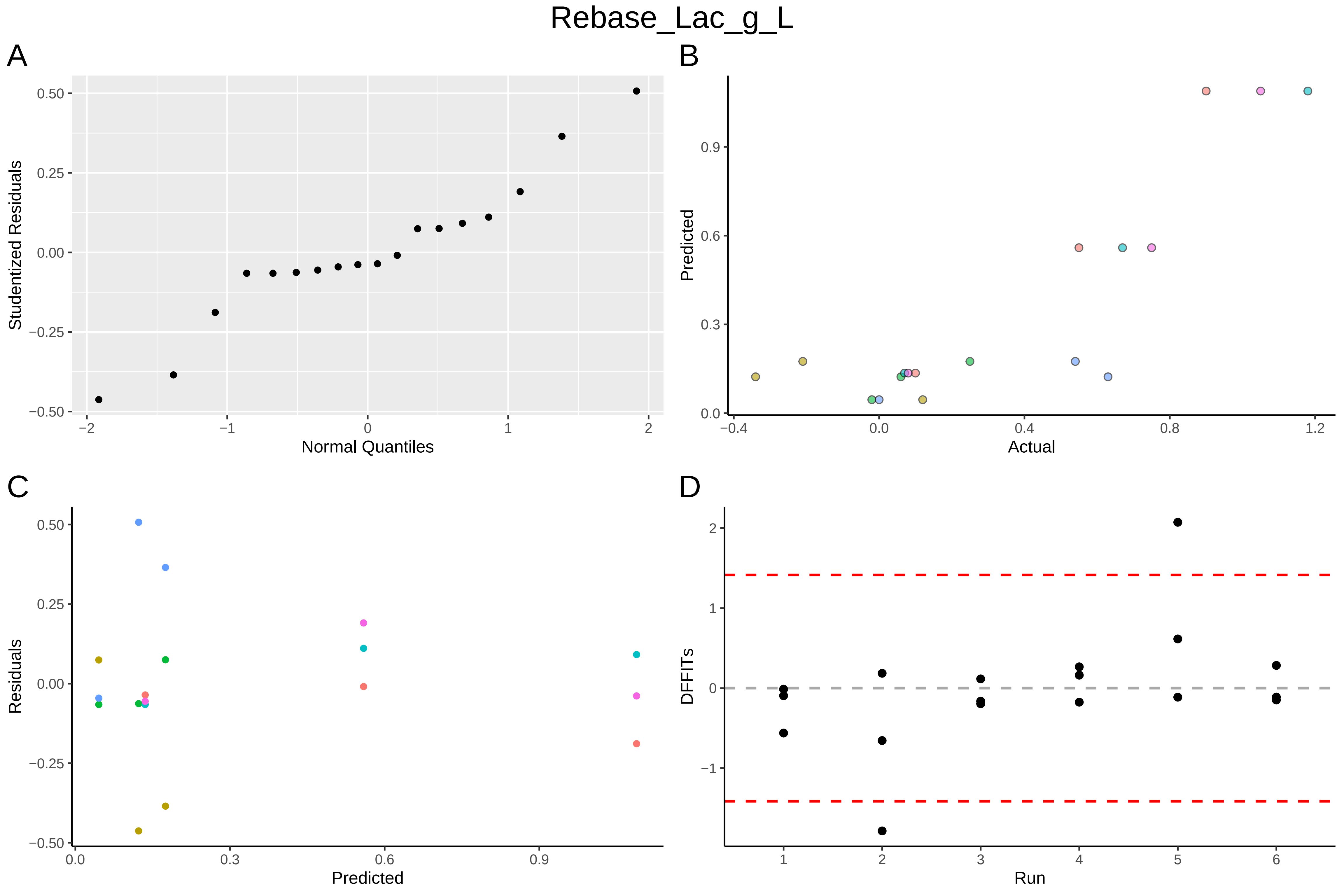

Rebase_Lac_g_L
A
Studentized Residuals
Normal Quantiles
0.50
0.25
0.00
−0.25
−0.50
−2
−1
0
1
2
B
Predicted
Actual
0.9
0.6
0.3
0.0
−0.4
0.0
0.4
0.8
1.2
C
Residuals
Predicted
0.50
0.25
0.00
−0.25
−0.50
0.0
0.3
0.6
0.9
D
DFFITs
Run
2
1
0
−1
1
2
3
4
5
6
