# Supplementary material for: Applying intensified design of experiments to mammalian cell culture processes
Source: Eng Life Sci. 2021 Nov 24;22(12):784–95. doi: 10.1002/elsc.202100123 (PMC9731596; doi:10.1002/elsc.202100123)
Supplement: Supplementary file 3 — Supporting information. [file ELSC-22-784-s002.pdf]

iDoE-Data Based OLS Model Coefficients for the Different Stages During Growth Phase

		Stage 1						Stage 2						Stage 3					
**Output**	**Terms**	**β**	**SE**	**SoS**	**Df**	***F***	***p***	**β**	**SE**	**SoS**	**Df**	***F***	***p***	**β**	**SE**	**SoS**	**Df**	***F***	***p***
	Time	4.41	0.34	0.45	1	163.99	< 1.00E-05	6.91	0.32	17.85	1	466.73	< 1.00E-05	9.06	0.5	55.82	1	327.56	< 1.00E-05
	Temperature	1.47	0.08	0.94	1	339.3	< 1.00E-05	2.18	0.14	9.34	1	244.27	< 1.00E-05						
	DO							-0.18	0.07	0.25	1	6.5	2.55E-02						
	Time^{2}	1.6	0.25	0.12	1	42.37	< 1.00E-05	3.04	0.87	0.47	1	12.23	4.41E-03						
	Temperature^{2}	0.08	0.03	0.02	1	8.1	9.16E-03	0.86	0.12	1.98	1	51.73	1.00E-05						
	DO^{2}							-0.68	0.09	2.2	1	57.49	1.00E-05						
	Time × Temperature	2.23	0.16	0.52	1	188.65	< 1.00E-05												
	DO × Temperature							-1.33	0.16	2.81	1	73.35	< 1.00E-05						
	Time × DO^{2}							-2.73	0.45	1.4	1	36.59	6.00E-05						
TCD	Time × Temperature^{2}													2.18	0.71	1.63	1	9.54	1.03E-02
	Time^{2} × Temperature													3.9	0.48	11.16	1	65.47	1.00E-05
	Time x DO x Temperature													-1.04	0.23	3.41	1	20.01	9.40E-04
	Time^{2} × DO^{2}													4.01	0.53	9.8	1	57.49	1.00E-05
	Time^{3} × Temperature							77.65	6.77	5.03	1	131.52	< 1.00E-05						
	Time x DO x Temperature^{2}							1.66	0.45	0.52	1	13.61	3.10E-03						
	Time^{2} x DO^{2} × Temperature													-5.65	1.26	3.44	1	20.19	9.10E-04
	Time^{3} × DO × Temperature							-43.95	7.57	1.29	1	33.68	8.00E-05						
	Time^{4} × Temperature	0.8	0.1	0.19	1	69.67	< 1.00E-05												
	Residuals			0.06	23					0.46	12					1.87	11		
	Time	11.42	3.52	0.02	1	10.52	3.88E-03	6.43	0.32	13.75	1	397.09	< 1.00E-05	8.94	0.5	54.34	1	315.94	< 1.00E-05
	Temperature	1.44	0.07	0.91	1	432.33	< 1.00E-05	2.14	0.13	9.02	1	260.44	< 1.00E-05						
	DO							-0.17	0.07	0.24	1	6.97	2.16E-02						
	Time^{2}	12.96	5.66	0.01	1	5.24	3.25E-02	3.03	0.83	0.46	1	13.42	3.25E-03						
	Temperature^{2}	0.09	0.02	0.03	1	13.03	1.64E-03	0.86	0.11	1.95	1	56.39	1.00E-05						
	DO^{2}							-0.69	0.09	2.23	1	64.31	< 1.00E-05						
	Time × Temperature	2.19	0.14	0.5	1	236.67	< 1.00E-05												
	DO × Temperature							-1.3	0.15	2.68	1	77.4	< 1.00E-05						
	Time × DO^{2}							-2.29	0.39	1.21	1	35.06	7.00E-05						
	Time × Temperature^{2}							1.62	0.43	0.49	1	14.28	2.63E-03	2.14	0.71	1.57	1	9.13	1.16E-02
VCD	Time^{2} × Temperature													3.85	0.48	10.92	1	63.48	1.00E-05
	Time x DO x Temperature													-1.04	0.23	3.44	1	20.01	9.40E-04
	Time^{3}	5.66	2.82	0.01	1	4.04	5.74E-02												
	Time^{2} × DO^{2}													3.93	0.53	9.42	1	54.79	1.00E-05
	Time^{3} × Temperature							76.76	6.44	4.92	1	141.93	< 1.00E-05						
	Time x DO x Temperature^{2}	-0.03	0.02	0.01	1	3.76	6.60E-02												
	Time^{3} × DO × Temperature							-43.41	7.21	1.26	1	36.29	6.00E-05						
	Time^{4} × Temperature	0.78	0.08	0.18	1	86.75	< 1.00E-05												
	Time^{2} x DO^{2} × Temperature													-5.59	1.26	3.37	1	19.59	1.02E-03
	Residuals			0.04	21					0.42	12					1.89	11		
	Time													-3.51	1.54	0.21	1	5.17	3.93E-02
	Time^{2}													2.15	1.09	0.16	1	3.84	7.02E-02
	Temperature^{2}							0.55	0.15	1.21	1	13.06	1.63E-03						
Viability	DO^{2}							-0.39	0.14	0.74	1	7.98	1.02E-02						
	Time × DO^{2}	0.54	0.23	1.05	1	5.36	2.81E-02												
	Time^{2} × Temperature													-0.19	0.1	0.14	1	3.58	7.92E-02
	Residuals			5.47	28					1.95	21					0.57	14		
	Time	7.43	0.68	1.83	1	120.6	< 1.00E-05	1.24	0.19	1.71	1	40.81	1.00E-05						
	Temperature	-0.73	0.1	0.89	1	58.65	< 1.00E-05	-1.21	0.15	2.91	1	69.38	< 1.00E-05						
	Time^{2}	4.91	0.48	1.58	1	104.24	< 1.00E-05												
	Temperature^{2}													0.24	0.07	0.18	1	11.94	4.76E-03
	DO^{2}							0.3	0.09	0.46	1	10.9	4.51E-03						
	Time × Temperature	-0.75	0.12	0.54	1	35.95	< 1.00E-05	-1.46	0.3	1	1	23.89	1.60E-04	-0.49	0.22	0.07	1	4.81	4.88E-02
	DO × Temperature							0.83	0.16	1.1	1	26.25	1.00E-04						
Glucose	Time × Temperature^{2}	0.09	0.04	0.07	1	4.53	4.43E-02												
	Time^{2} × Temperature													-0.03	0.27	0	1	0.01	9.24E-01
	Time^{2} × DO							2.16	1.08	0.17	1	4.04	6.17E-02						
	DO^{2} × Temp^{2}	-0.14	0.05	0.12	1	7.73	1.06E-02												
	Time^{2} × Temperature^{2}							-5.66	1.62	0.51	1	12.23	2.98E-03						
	Time^{3} x DO^{2}													-0.63	0.1	0.56	1	37.34	5.00E-05
	Time^{4} × DO × Temperature													0.24	0.08	0.12	1	7.81	1.62E-02
	Residuals			0.35	23					0.67	16					0.18	12		
	Time	-24.43	9.83	0	1	6.17	2.19E-02	2.03	0.12	4.64	1	305.18	< 1.00E-05	1.56	0.33	1.39	1	22.17	3.40E-04
	Temperature	0.66	0.02	0.19	1	740.53	< 1.00E-05	0.55	0.09	0.61	1	40.13	1.00E-05						
	Time^{2}	-63.4	22.53	0	1	7.92	1.07E-02												
	DO^{2}							-0.12	0.05	0.07	1	4.74	4.30E-02	0.07	0.21	0.01	1	0.1	7.56E-01
	Time × Temperature	0.83	0.05	0.07	1	276.42	< 1.00E-05	0.72	0.18	0.25	1	16.26	7.80E-04						
	DO × Temperature							-0.25	0.1	0.1	1	6.38	2.11E-02						
Lactate	Time^{3}	-65.88	21.91	0	1	9.04	6.96E-03												
	Time^{4}	-24.31	7.71	0	1	9.94	5.00E-03												
	Time^{2} × DO^{2}													-1.03	0.33	0.6	1	9.65	7.72E-03
	DO^{2} × Temperature^{2}	0.1	0.02	0.01	1	26.12	5.00E-05												
	Time x DO^{2} × Temperature^{2}	0.11	0.03	0	1	18.53	3.40E-04												
	Time^{4} × Temperature	0.17	0.03	0.01	1	33.91	1.00E-05												
	Residuals			0.01	20					0.27	18					0.88	14		

Abbreviations: iDoE = intensified design of experiments, OLS = ordinary least squares, TCD = total cell density, VCD = viable cell density, β = coefficient estimate, SE = standard error, DO = dissolved oxygen, SoS = sum of squares, Df = degrees of freedom

Model Qualities for DoE-Data Based OLS Regressions

Read Out	R^2	R^2_{adj}	R^2_{pred}	RMSE	SE	F	df_{num}	df_{den}
TCD	0.99	0.99	0.99	0.02	0.02	2166.13	6	69
VCD	0.99	0.99	0.99	0.02	0.02	1510.45	8	67
Viability	0.40	0.35	0.15	0.11	0.12	7.82	6	69
Glucose	0.97	0.97	0.96	0.04	0.04	270.29	8	67
Lactate	0.98	0.98	0.98	0.05	0.05	442.18	9	66

Coefficients of DoE-Based OLS Models for the Growth Phase

Response	Model Terms	β	SE	SoS	Df	*F*	*p*
TCD	Time	0.300	< 0.0001	2.98	1	7307.8	< 0.0001
	Temperature	0.140	0.010	0.04	1	91.2	< 0.0001
	$Time^2$	0.150	0.010	0.26	1	642.3	< 0.0001
	Time × Temperature	0.180	< 0.0001	0.74	1	1826.0	< 0.0001
	$Temperature^3$	-0.060	0.010	0.01	1	15.8	1.70E-04
	$Time^2$ × Temperature	0.090	0.010	0.06	1	157.0	< 0.0001
	Residuals			0.03	69		
VCD	Time	0.300	< 0.0001	2.96	1	6780.9	< 0.0001
	Temperature	0.140	0.020	0.03	1	78.0	< 0.0001
	$Time^2$	0.150	0.010	0.26	1	587.2	< 0.0001
	Time × Temperature	0.180	< 0.0001	0.74	1	1704.6	< 0.0001
	$Temperature^3$	-0.040	0.020	0.00	1	4.6	3.66E-02
	$Time^2$ × Temperature	0.090	0.010	0.06	1	141.1	< 0.0001
	DO^2 × Temperature	-0.020	0.010	0.00	1	2.7	1.04E-01
	Time x DO x Temperature	-0.010	< 0.0001	0.00	1	2.5	1.19E-01
	Residuals			0.03	67		
Viability	Time	-0.070	0.050	0.04	1	2.6	1.14E-01
	$Time^2$	-0.120	0.030	0.15	1	11.1	1.40E-03
	$Time^2$ × Temperature	-0.130	0.050	0.11	1	7.6	7.39E-03
	$Temperature^2$	-0.110	0.030	0.16	1	11.5	1.15E-03
	DO^2	0.090	0.030	0.10	1	7.3	8.62E-03
	Time × DO^2	0.160	0.050	0.16	1	11.4	1.22E-03
	Residuals			0.95	69		
Glucose	Time	0.210	0.020	0.20	1	126.4	< 0.0001
	Temperature	-0.260	0.030	0.13	1	78.0	< 0.0001
	DO	0.010	0.010	0.00	1	2.9	9.33E-02
	$Time^2$	-0.110	0.010	0.14	1	88.7	< 0.0001
	Time × Temperature	-0.240	0.010	1.30	1	812.6	< 0.0001
	$Time^3$	-0.180	0.020	0.10	1	63.7	< 0.0001
	$Temperature^3$	0.090	0.030	0.01	1	9.1	3.68E-03
	$Time^2$ × DO	-0.040	0.010	0.01	1	7.6	7.44E-03
	Residuals			0.11	67		
Lactate	Time	0.750	0.030	1.92	1	799.4	< 0.0001
	Temperature	0.210	0.010	0.94	1	390.7	< 0.0001
	DO	-0.010	0.010	0.01	1	4.0	5.09E-02
	$Time^2$	-0.060	0.010	0.04	1	16.2	1.50E-04
	$Temperature^2$	-0.030	0.010	0.01	1	4.8	3.15E-02
	Time × Temperature	0.050	0.010	0.07	1	28.2	< 0.0001
	$Time^3$	-0.290	0.030	0.27	1	113.7	< 0.0001
	$Time^2$ × DO	-0.200	0.020	0.29	1	120.0	< 0.0001
	Time × $Temperature^2$	-0.060	0.020	0.02	1	9.9	2.53E-03
	Residuals			0.16	66		

Abbreviations: DoE = design of experiments, OLS = ordinary least squares, TCD = total cell density, VCD = viable cell density, β = model term coefficient, SE = standard error, SoS = sum of squares, Df = degrees of freedom, DO = dissolved oxygen
